# Critical View of Novel Treatment Strategies for Glioblastoma: Failure and Success of Resistance Mechanisms by Glioblastoma Cells

**DOI:** 10.3389/fcell.2021.695325

**Published:** 2021-08-16

**Authors:** Timo Burster, Rebecca Traut, Zhanerke Yermekkyzy, Katja Mayer, Mike-Andrew Westhoff, Joachim Bischof, Uwe Knippschild

**Affiliations:** ^1^Department of Biology, School of Sciences and Humanities, Nazarbayev University, Nur-Sultan, Kazakhstan; ^2^Department of General and Visceral Surgery, Surgery Center, Ulm University Hospital, Ulm, Germany; ^3^Department of Pediatrics and Adolescent Medicine, University Medical Center Ulm, Ulm, Germany

**Keywords:** glioblastoma, immunotherapy, immune evasion, tumor microenvironment, peptide and mRNA vaccines

## Abstract

According to the invasive nature of glioblastoma, which is the most common form of malignant brain tumor, the standard care by surgery, chemo- and radiotherapy is particularly challenging. The presence of glioblastoma stem cells (GSCs) and the surrounding tumor microenvironment protects glioblastoma from recognition by the immune system. Conventional therapy concepts have failed to completely remove glioblastoma cells, which is one major drawback in clinical management of the disease. The use of small molecule inhibitors, immunomodulators, immunotherapy, including peptide and mRNA vaccines, and virotherapy came into focus for the treatment of glioblastoma. Although novel strategies underline the benefit for anti-tumor effectiveness, serious challenges need to be overcome to successfully manage tumorigenesis, indicating the significance of developing new strategies. Therefore, we provide insights into the application of different medications in combination to boost the host immune system to interfere with immune evasion of glioblastoma cells which are promising prerequisites for therapeutic approaches to treat glioblastoma patients.

## Introduction

Glioblastoma is the most invasive and therapy-insensitive type of glial tumor. The subpopulation of glioblastoma cells is disposed to proliferate quickly and in an uncontrolled manner, where alternative subsets infiltrate into nearby healthy tissue making an entire resection impossible. The high heterogeneity of glioblastoma contributes to tumor progression and recurrence which causes resistance from therapeutic drugs (Vieira de Castro et al., 2020; Zhang and Liu, 2020). On the molecular level, glioblastoma cells, for instance, often fail the proper regulation of pro- and anti-apoptotic proteins leading to apoptotic resistance (Vengoji et al., 2018). Additionally, glioblastoma stem cells (GSCs), using altered signaling pathways, do not only play a critical role in resistance to conventional therapies and tumor recurrence, but also in tumor initiation and progression (Dean et al., 2005; Vengoji et al., 2018; Bhaduri et al., 2020; Vieira de Castro et al., 2020). Hypoxia is a prominent microenvironmental factor that results from a rapid growth of glioblastoma and the need for oxygen, provoking angiogenesis as well as anaerobic glycolysis. This, in turn, generates a local accumulation of lactate with a decreased pH environment and encourages glioblastoma cells for migration (Xie et al., 2014).

The aim of the review is to provide a critical overview of the common as well as novel treatment strategies of glioblastoma based on the resistant mechanisms developed by glioblastoma cells and to ascertain combination therapies to improve the failure of monotherapies.

### Standard Care Therapies for Glioblastoma

**FIGURE 1:**
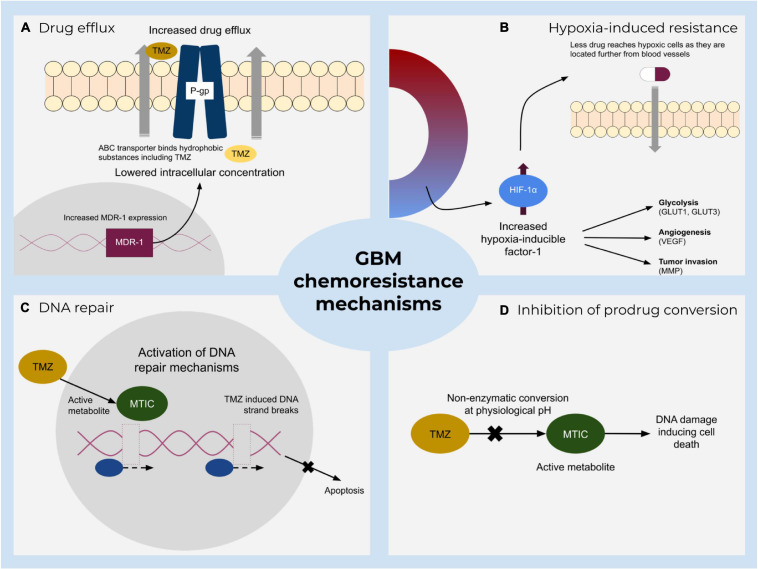
A summary of major resistance mechanisms to standard treatment of glioblastoma. Main impeding factors include **(A)** increased active TMZ efflux by ATP-binding cassette transporters that lower the drug concentration and hence its effect. **(B)** Hypoxia-mediated chemoresistance caused by various hypoxia-inducible factor-1 (HIF-1) activities. **(C)** Direct and indirect DNA damage repair mechanisms and **(D)** inhibition of prodrug conversion which prevents DNA damage.

Standard of care therapies for glioblastoma include resection, radio- and chemotherapy with the administration of temozolomide (TMZ) or nitrosoureas components. Chemoresistance during medication is a major drawback and includes several mechanisms (Figure 1), such as drug metabolic inactivation, increased DNA-repair mechanisms, inhibition of prodrug conversion, and lowering the intracellular drug concentration by enhanced drug efflux via enhanced expression of transporters (Wee et al., 2016; Sharifzad et al., 2019; Pessina et al., 2020). TMZ is frequently described as a DNA alkylating prodrug that induces double-strand breaks of the DNA which eventually lead to apoptosis (Karachi et al., 2018; Sharifzad et al., 2019). Recently, a debate has emerged casting doubt on the precise molecular function of this component (Strobel et al., 2019; Kaina, 2019; Stepanenko and Chekhonin, 2019; Westhoff et al., 2020; Herbener et al., 2020). According to the traditionally proposed model, TMZ alkylates bases that are present in the DNA, leading to a mismatch during replication and induces futile rounds of DNA repair, finally ending in DNA strand breaks and apoptosis (Kaina, 2019). The O^6^-methylguanine-DNA-methyltransferase (MGMT) can resolve some of the TMZ-induced alterations and thus mediate survival, but is frequently found not to be expressed in approximately half of all glioblastoma cells, i.e., about 45% of patients considered to benefit from TMZ (Karachi et al., 2018; Miyauchi and Tsirka, 2018; Arora and Somasundaram, 2019). The standard practice, however, is still to prescribe TMZ treatment notwithstanding a patient’s methylation level (Kamson and Grossman, 2021), although studies show no statistically significant difference in survival between the groups controlling for TMZ in the absence of MGMT methylation (Hegi et al., 2005). Indeed, even the predictive value of MGMT promotor methylation with regards to tumor response to TMZ is not uncontroversial (Stepanenko and Chekhonin, 2019; Yu et al., 2020). Overall, TMZ treatment extends patient survival from 12.1 to 14.6 months (Strobel et al., 2019), i.e., modulating its therapeutic potency is unlikely to be curative, but might further extend the therapeutic window.

MGMT inhibitors are therefore considered to be of interest to improve the clinical response to TMZ treatment (Karachi et al., 2018; Arora and Somasundaram, 2019). However, systemic inhibition of MGMT might lead to increased apoptosis or even the accumulation of mutations in healthy tissue, so that localized application of inhibiting pseudo-substrates or tumor-specific delivery of blocking peptides have been considered as strategies to increase the efficiency of TMZ treatment while not concurrently sensitizing healthy tissue to the alkylating agent (Yu et al., 2020; Wängler et al., 2020, respectively). Some of these strategies are currently being evaluated clinically and are showing rather promising results (Yu et al., 2020). Alternatively, metronomic application of TMZ might suffice to sensitize relatively resistant glioblastoma cells to this drug, as MGMT is a suicide enzyme that is destroyed upon de-alkylating a base (Karachi et al., 2018; Miyauchi and Tsirka, 2018; Arora and Somasundaram, 2019; Le Rhun et al., 2019; Strobel et al., 2019).

The treatment of recurrent glioblastoma encompasses nitrosourea compounds, namely lomustine (Brandes et al., 2016; Le Rhun et al., 2019). Nitrosoureas are alkylating reagents which are able to cross the blood-brain barrier due to their high lipophilicity, causing cell damage and apoptosis. However, lomustine implies severe adverse reactions (prolonged thrombocytopenia and dose-limiting pulmonary toxicity was determined) (Brandes et al., 2016). The small increase of overall survival (OS) while the progression-free survival (PFS) for newly diagnosed as well as recurring glioblastoma denotes the need for better management of the disease by reducing severe side effects and preventing resistance.

### Glioblastoma Cells Are Resistant to Apoptosis

**FIGURE 2:**
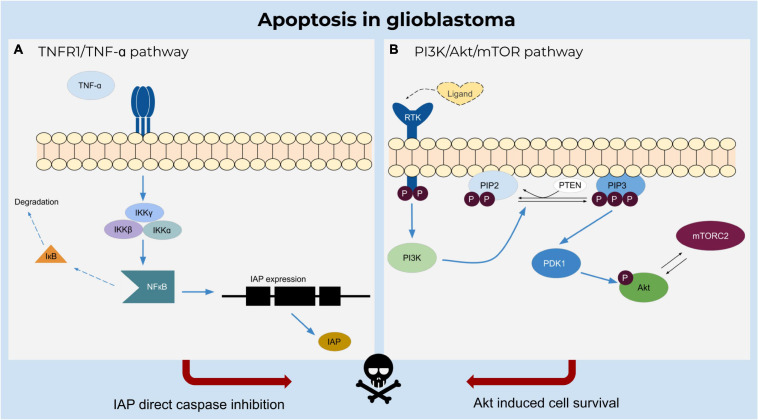
Apoptosis in glioblastoma. **(A)** TNF-α induces inhibition of caspases by activating NFκB upon binding to TNFR1 receptor and triggering the IKK complex to degrade the inactivating factor IκB. NFκB regulates the expression of IAPs which inhibit apoptosis. **(B)** Ligand binding to the receptor tyrosine kinase induces catalytic activity of PI3K and further phosphorylates PIP2 to PIP3. The phosphorylation of the Akt molecule is mediated by PDK1 and activated by PIP3, resulting in a cascade of cell survival.

Apoptosis plays an important role in the elimination of damaged cells in multicellular organisms to sustain normal biological processes (Figure 2). A dysregulation of apoptosis can lead to cancer and other pathophysiological disorders. In the case of preventing apoptosis or establishing resistance, anti-apoptotic proteins (e.g., B cell lymphoma-2 protein family members Bcl-2 or Bcl-xL) bind to pro-apoptotic family members (e.g., Bcl-2 homologous antagonist/killer (Bak) or Bax) resulting in the neutralization of their activity (Tsujimoto, 1998; Ola et al., 2011).

Another attractive target for sensitization to therapy is the Bcl-2 family, including Bcl-2, Bcl-xL and Bcl-w, which inhibit the release of mitochondrial apoptogenic factors by sequestering Bak and Bax (Strik et al., 1999; Wick et al., 2004; Stegh et al., 2007). Apart from overexpression of anti-apoptotic proteins, the downregulation of pro-apoptotic proteins (Bak, Bax, Box, and NOXA) has been described for glioblastoma (Strik et al., 1999; Tyagi et al., 2002; Steinbach and Weller, 2004). Low protein levels in glioblastoma patients have been detected for Apaf-1 and procaspase-9 (Blahovcova et al., 2015). Consequently, the initiation of apoptosis is circumvented and therapeutic response is poor (Tagscherer et al., 2008). The Bcl-2 family proteins act as a critical regulator of life-death decisions within the apoptotic pathway and especially the inactivation or downregulation of anti-apoptotic Bcl-2 family members, represent an interesting target for anticancer therapies. The expression and activity of the Bcl-2 protein can be decreased by using antisense oligonucleotides, small molecules, or peptides (Ola et al., 2011). Antisense oligonucleotides, for instance Genasense (not approved by FDA), lead to the degradation of *bcl-2* mRNA or incites a steric hindrance of translation, which reduces Bcl-2 expression (Julien et al., 2000; Frankel, 2003). Small molecule inhibitors, such as ABT-737, ABT-263 (navitoclax), or more recently the FDA approved alternative ABT-199 (venetoclax), BH3 mimetics, were designed to block the BH3 domain binding site on the surface of Bcl-2 and/or Bcl-xL, preventing inhibition of pro-apoptotic Bak or Bax (An et al., 2004; Kouri et al., 2012; Singh et al., 2019). Interestingly, in the context of glioblastoma, navitoclax has been the more promising substance, suggesting that modulation of Bcl-xL is needed for therapeutic efficacy (Hlavac et al., 2019; Nguyen et al., 2019), unfortunately navitoclax is also associated with serious, but manageable side effects, such as thrombocytopenia and neutropenia, severely limiting its therapeutic value (Wilson et al., 2010; Kuter, 2015). Additionally, the anti-apoptotic Bcl-2 family member myeloid cell leukemia factor-1 (Mcl-1), which interferes in early cascade events by suppressing cytochrome c release from mitochondria, is highly expressed in human glioblastoma (Michels et al., 2005; Karpel-Massler et al., 2017). High levels of Mcl-1 mediate BH3-mimetic resistance, suggesting that a dual inactivation of Bcl-2/Bcl-xL and Mcl-1 is necessary for apoptosis of glioblastoma cells (Kouri et al., 2012; Karpel-Massler et al., 2017; Shang et al., 2020). AT-101, a small molecule targeting Mcl-1, is presently being examined in a clinical trial (Xiang et al., 2018). Although inhibition of Mcl-1 has received certain attention as a potential drug target, only slowly implemented in clinical applications and poses challenges in glioblastoma treatment due to high molecular weight and the blood brain barrier. With the aim to overcome such drawbacks the use of combinatory approaches is further explored (Tron et al., 2018; Shang et al., 2020).

In order to circumvent apoptosis resistance of glioblastoma, inhibitor of apoptosis (IAP) proteins can also be targeted by small molecule inhibitors resulting in enhanced sensitivity to radiation and TRAIL (Vellanki et al., 2009; Lincoln et al., 2018).

Regarding the death receptor-mediated pathways, gene expression of several members of the TNF receptor family as well as FAS and FADD but also caspase-8 and caspase-7 was found to be reduced in human glioblastoma tissue. Subsequently, DISC formation and initiation of apoptosis by both the death receptor and mitochondrial pathway are affected favoring resistance to apoptosis and different therapies (Blahovcova et al., 2015; Wang et al., 2017). As an approach to sensitize glioblastoma cells to TRAIL-induced cell death, lanatoside C has been shown to upregulate expression of TRAIL-R2 as well as to activate a necrosis-like and caspase-independent cell death pathway. Low doses of lanatoside C demonstrated significant anti-glioblastoma activity in cell culture and glioblastoma xenografts when combined with low doses of TRAIL (Badr et al., 2011). However, even if the expression of TRAIL-R1 and TRAIL-R2 is increased in glioblastoma cells, resistance to TRAIL-induced apoptosis can be caused by low levels of caspase-8 and FADD (Knight et al., 2001).

## The Tumor Microenvironment Provokes Resistance

Advances in immunotherapies are promising approaches for an efficient management of glioblastoma. However, resistance mechanisms to immunotherapy and poor understanding of the glioblastoma microenvironment are still a major drawback for the development of novel therapies (Razavi et al., 2016; Adhikaree et al., 2020).

### The Immune System in the Brain

The idea of an immune privileged location of the brain is challenged by the facts that the CNS possesses a lymphatic vessel network, which can attract leukocytes to migrate from the cerebrospinal fluid to the cervical lymphatics and back. These leukocytes can traverse to the CNS even with an intact blood-brain barrier (Li et al., 2001; Razavi et al., 2016), which offers new perspectives for immunotherapy. Besides that, inflammation increases the permeability of the blood-brain barrier enabling the intrusion of circulating monocytes and lymphocytes into the brain (Sharifzad et al., 2019; Adhikaree et al., 2020). In particular, the blood-brain barrier is disrupted in glioblastoma, increasing the numbers of immune cells in the CNS (Amin et al., 2012) by compromised tight junctions and degradation of proteoglycans of the extracellular matrix by proteases (Schneider et al., 2004). Furthermore, glioblastoma is known as a highly vascularized tumor with vessels having a larger diameter. Pericytes, which normally surround endothelial cells, are sparse along these vessels, thereby expanding the leakiness of the blood-brain barrier (Miyauchi and Tsirka, 2018).

Professional antigen-presenting cells (APCs), like dendritic cells (DCs), can display exogenous tumor antigens on major histocompatibility complex class I (MHC I) or II molecules, priming CD8^+^ T cells (cytotoxic T lymphocytes, CTLs) or CD4^+^ T cells (T helper cells, Th), respectively. IFN-γ signaling is responsible for the upregulation of MHC I and MHC II molecules on target cells, allowing an increased ability to present antigenic peptides on MHC for T cell inspection and amplify an anti-tumor specific immune response by migrating into the brain parenchyma (Engelhardt and Ransohoff, 2012; Candeias and Gaipl, 2016). Contrastingly, reduced levels of MHC I molecules are found on glioblastoma cells, depending on the patient sample or cell lines investigated, which represents an immune evasion mechanism of glioblastoma cells (summarized in Burster et al., 2021).

### The Tumor Microenvironment and Immune Evasion

**FIGURE 3:**
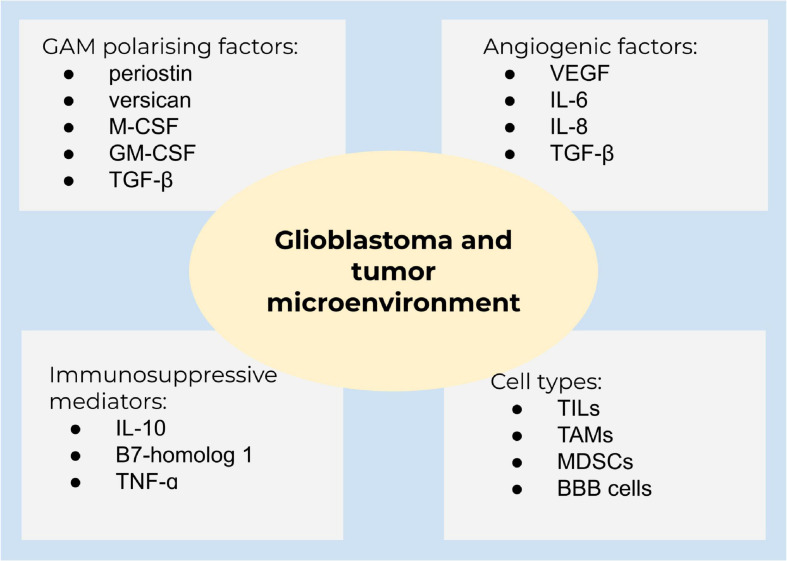
Glioblastoma and the tumor microenvironment. A summary of main components of the tumor microenvironment in glioblastoma.

The tumor microenvironment is surrounded by cytokines, chemokines, and growth factors to recruit immune cells to enable tumor growth and progression, seriously affecting the therapeutic cure (Figure 3). Examples for cells in this niche are B cells and T cells, such as T regulatory cells (Tregs), so-called tumor-infiltrating lymphocytes (TILs), tumor-associated macrophages (TAMs), myeloid-derived suppressor cells (MDSCs), and blood-brain barrier cells, which communicate by secreted mediators to facilitate cell growth, invasion, therapeutic resistance, and immune evasion (Ou et al., 2020).

Glioblastoma is characterized by the growth of new capillaries from existing blood vessels, containing hyperpermeable vessels with an enlarged diameter. The vasculature in solid tumors ensures tumor growth and sufficient oxygen and nutrient supply (Weathers and de Groot, 2015; Ahir et al., 2020). The high vascularization is caused by the expression of angiogenetic factors, including VEGF, interleukin 6 (IL-6), IL-8, and transforming growth factor-β (TGF-β). Clinical studies demonstrated that the abundance of glioma-associated microglia/macrophages (GAMs) is higher in high-grade gliomas compared to low-grade gliomas (De et al., 2016). GAMs are polarized into an immunosuppressive state by TGF-β, periostin, versican, macrophage colony-stimulating factor (M-CSF), and granulocyte-macrophage colony-stimulating factor (GM-CSF). Immunosuppressive mediators, such as IL-10 and B7-homolog 1 (B7-H1), are highly expressed in glioblastoma. Chemoattractants [*inter alia*, hepatocyte growth factor (HGF), TGF-β, and granulocyte-colony stimulating factor (G-CSF)] attract different cell populations to the tumor microenvironment and lead from a decreased pro-inflammatory to an amplified anti-inflammatory state (Hanahan and Weinberg, 2011; De et al., 2016). Macrophages and microglia are able to switch between two distinctive phenotypes, the M1 pro-inflammatory and the M2 cytoprotective and immunosuppressive subpopulation, contributing to tumor cell proliferation, migration, and invasion. M2 are subdivided to M2a, M2b, and M2c due to the functional activation state. Of these, the M2c subset is dominant in gliomas (Gabrusiewicz et al., 2016). M2 macrophages produce immunosuppressive cytokines (TGF-β and IL-10), activating M2c (Gieryng et al., 2017), and are well-known to regulate T cell function (Wu et al., 2010). GAMs are recruited to the tumor site and being polarized into the M2 phenotype by different mediators, for instance, M-CSF and GM-CSF (Pyonteck et al., 2013). In addition, TGF-β isoform 1 and 2, prostaglandin E2, and the extracellular matrix protein periostin are involved in M2 polarization (Strepkos et al., 2020). Accordingly, TGF-β2 stimulates the synthesis of the proteoglycan versican by glioblastoma cells, which promotes GAM-induced inflammatory cytokine production to enhance glioma invasion (Arslan et al., 2007; Strepkos et al., 2020). GSCs also secrete factors to polarize GAMs to M2 and impede phagocytosis of M1 (Wu et al., 2010). Accordingly, targeting the interaction of cytotoxic and apoptotic effects of M2 receptor activation, several preclinical and phase I studies are currently on the way to promote GAM M1-like polarization or alter the microglia polarization, including emactuzumab, plerixafor, and maraviroc (Di Bari et al., 2015; Mercurio et al., 2016; Laudati et al., 2017). Nevertheless, the sophisticated crosstalk of tumor microenvironment and tumor heterogeneity of glioblastoma significantly contribute to therapy resistance (Strepkos et al., 2020).

The heterogenous population of immune cells from the myeloid lineage, MDSCs, are important for tumor survival within the tumor microenvironment. The amount of these cells is higher in the glioblastoma tumor microenvironment and suppress cytotoxic NK cell, CD4^+^- and CD8^+^ T cell function (Marvel and Gabrilovich, 2015; Gieryng et al., 2017). Two subsets of MDSCs exist: Polymorphonuclear- and mononuclear-MDSCs. Both use different mechanisms to suppress CD8^+^ T cells. Polymorphonuclear-MDSCs, predominantly found in glioblastoma, produce high levels of reactive oxygen species, cross-talk with Tregs, and secret immunosuppressive cytokines. Mononuclear-MDSCs express, for instance, nitric oxide synthase 2 and arginase (ARG1), which inhibit T cell proliferation and further promote T cell apoptosis (Marvel and Gabrilovich, 2015; Gieryng et al., 2017).

NK cells are important for killing cancer cells (Gieryng et al., 2017). However, NK cells are found to be impaired in the glioblastoma tumor microenvironment since these cells express a limited amount of MHC I molecules, binding to inhibitory NK cell receptors, and quench NK cell activation (Wiendl et al., 2002; Gieryng et al., 2017; Burster et al., 2021). The inhibitory receptors are isoforms of the killer-cell Ig-like receptors (KIR) and Ig-like transcript/leukocyte Ig-like receptors (ILT/LIR). Moreover, TGF-β1 downregulated the expression of activating NK cell receptors, e.g., NKG2D and NKp30, thus inhibiting NK cell function (Castriconi et al., 2003). Additionally, TGF-β represses the mammalian target of the rapamycin (mTOR) pathway, which in turn reduces the proliferation and metabolic activity of NK cells (Viel et al., 2016).

Recruitment of non-neoplastic cells to the tumor microenvironment is implemented by glioblastoma. GAMs and glioblastoma cells express hepatocyte growth factor/scatter factor (HGF/SF), which binds to c-Met tyrosine kinase and promotes proliferation and invasion of glioblastoma cells (Kunkel et al., 2001). Monocyte chemotactic protein 1 (MCP-1 also named CCL2) and 3 are responsible for cell migration, for example, the binding of MCP-1 to CCR2 direct immune cells to infiltrate the tissue (Strepkos et al., 2020). This is in contrast to microglia which are attracted by a glial-derived neurotrophic factor (GDNF) expressed by neurons and glial cells (Ku et al., 2013).

Immune evasion by glioblastoma cells is achieved via immune checkpoint molecules. The programmed death-ligand 1 (PD-L1), an immune checkpoint molecule, is expressed on APCs, NK cells, parenchymal cells, and glioblastoma cells (Huang et al., 2017). After binding of PD-L1 to the PD-1 receptor, which is expressed on activated T cells, PD-L1-PD-1 receptor complex provokes T cell anergy or apoptosis (Razavi et al., 2016; Huang et al., 2017). Additional immune checkpoint molecules include cytotoxic T-lymphocyte-associated protein 4 (CTLA-4), T cell Ig, and mucin domain 3 (TIM-3) (Razavi et al., 2016). Furthermore, the immunomodulatory mechanism in glioblastoma is achieved through indolamine 2,3-dioxygenase 1, which is a cytosolic enzyme, inhibits T effector function and supports Treg expansion and activation, hence contributing to suppressing an immune response (Adhikaree et al., 2020).

## Immunotherapy and Limitation in Glioblastoma

Immunotherapy is based on the concept that the immune system is capable of recognizing and destroying tumor cells. However, the success of immunotherapy remains challenging since tumor cells developed various pathways to avoid being detected by the immune system, leading to low therapeutic efficiency. Glioblastoma adapted a high resistance and only a few patients respond to certain immunotherapies (Jackson et al., 2019).

### Antibody Drug Conjugates (ADCs)

EGFR gene amplification and mutations in the EGFR variant III (EGFRvIII) gene (Malkki, 2016; van den Bent et al., 2017) can be utilized for glioblastoma therapy by using antibody drug conjugates (ADCs). ADCs are monoclonal antibodies linked to different cytotoxic drug components (van den Bent et al., 2017). ADCs are designed to bind to tumor-specific antigens, which are internalized through receptor-mediated endocytosis. During this process, the cytotoxic components are released from the antibody due to the low pH environment of the endocytic compartment. While the receptor is recycled back to the cell surface, the delivered cytotoxic components induce apoptosis in tumor cells. One cavity of application is the fact that ADCs might be extracellularly released because of the proteolytic labile linker or ADCs are recycled back to the cell surface without delivering the cytotoxic component, leading to a reduced drug concentration in the cell (Polson et al., 2009; Peters and Brown, 2015; Scotti et al., 2015). The recycling mechanism of ADCs is caused by the high affinity of ADCs to neonatal Fc receptors (FcRns) within early endosomes that reach the cell surface recurrently, where ADCs are released from the FcRn under physiological pH. Notably, the FcRn is predominantly expressed in the endosomes of endothelial cells (Peters and Brown, 2015).

The ADCs are often DNA alkylating agents, inhibitors of tubulin polymerization, or enediyne antibiotics which lead to DNA double-strand breaks (Scotti et al., 2015). Limitations of ADC application is indicated by the restriction of available specific tumor-associated antigens (TAA), downregulation of TAAs, and the transport of ADCs to the brain (Phillips et al., 2016; Miyauchi and Tsirka, 2018). On the other hand, previous studies acknowledged the possible uptake of ADCs in patients by disrupted blood-brain barrier (Phillips et al., 2016). Of note, distinct glioblastoma patients have a relatively low mutational burden compared to non-small cell lung cancer, minimizing the amount of potential TAAs (Miyauchi and Tsirka, 2018). Lack of blood-brain barrier penetration, tumor heterogeneity, and resistance to the cytotoxic agent impede therapy success (Gieryng et al., 2017). Although a successful uptake of ADCs through the blood-brain barrier of low-grade gliomas with little disrupted blood-brain barrier compared to high-grade gliomas could undermine complete penetration due to the size of ADCs. High-grade gliomas exhibit augmented microvascular permeability, allowing penetration of larger molecules (Roberts et al., 2000; Schneider et al., 2004). Certainly, a specific antigen for targeting glioblastoma is needed; however, tumor heterogeneity or reduced levels of antigens are one of several reasons for therapy resistance (Gan et al., 2017). An additional limitation of this approach is an acquired resistance to the cytotoxic agent of ADC, namely active efflux of the internalized drug is possible through transporters of the adenosine triphosphate-binding cassette family (Scotti et al., 2015).

### Immune Checkpoint and Chemokine Inhibitors

Monoclonal antibodies, known as nivolumab, pembrolizumab, ipilimumab, and atezolizumab, are widely used in cancer treatment by interrupting checkpoint signaling pathways and leading to an immune-mediated elimination of tumor cells (Juszczak et al., 2012; Darvin et al., 2018). While the patients benefit from prolonged OS, distinct immune-related adverse reactions exist, including pneumonitis or lymphocytic hypophysitis (an inflammation of the pituitary gland). The use of monoclonal antibodies are limited and not suitable for patients which suffer from autoimmunity because immune checkpoints in general act as barriers against autoimmune disorders (Juszczak et al., 2012). Approved immune checkpoint inhibitors are presently tested for primary and recurrent brain malignancies. The administration of nivolumab (anti-PD-1 monoclonal antibody) caused mild side effects, whereas nivolumab in combination with ipilimumab initiated severe (grade 3 and 4) adverse effects in 80% of the treated patients. Notably, 50% discontinued the medication due to intolerability. The 6-months OS rate for nivolumab was 75, 80% for the combination therapy. Currently, nivolumab in combination with bevacizumab is being tested in a phase III clinical trial (NCT02017717) (Sampson et al., 2015). Despite encouraging results in OS, only a limited number of patients benefit from monoclonal antibodies, as many tumors downregulate T cell activity or prevent T cells from infiltrating the tissue, indicating an urgent need for better predictive biomarkers (Darvin et al., 2018).

Chemokines are highly expressed in the tumor microenvironment and support tumor progression; therefore, blocking of chemokine receptors is a promising approach (Laudati et al., 2017). In a preclinical study, maraviroc, a CCR5 receptor blocker, polarized microglia toward the cytotoxic M1 phenotype by reducing the gene expression of ARG1 and IL-10, which are two M2 macrophage markers. Additionally, M1 markers are upregulated by inhibition of the mTOR pathway. The treatment with maraviroc, a small molecule CCR5 antagonist (Carter and Keating, 2007), led to a reduction of microglia migration (Laudati et al., 2017). Plerixafor, another small molecule CXCR4 antagonist, binds to the three acidic residues of the CXCR4 ligand-binding pocket (Fricker, 2008) and impairs the proliferation of glioma cells by inhibiting the invasion of CXCR4/CXCR7-expressing GSCs *in vitro* (Mercurio et al., 2016; Hira et al., 2017). Plerixafor, on the other hand, is not highly specific for CXCR4 as Plerixafor might bind to the receptor of CXCL12, CXCR4, and CXCR7. As a result, cardiotoxicity is one of the adverse reactions. Interestingly, a novel CXCR4 antagonist, Peptide R [Arg-Ala-(Cys-Arg-Phe-Phe-Cys), with the square brackets indicating cyclization *via* a disulfide bridge], reduced toxicity in mice (Portella et al., 2013; Mercurio et al., 2016).

Targeting STAT3, which is involved in maintaining an immunosuppressive environment in glioblastoma and is consistently activated in a variety of tumors, is an alternative therapeutic strategy for glioblastoma (See et al., 2012). The STAT3 inhibitor WP1066, also a small molecule, polarizes GAMs to a M1 cytotoxic phenotype and can block glioma growth *in vivo* (Hussain et al., 2007). A major challenge occurs with differences in the immune system between humans and animals; therefore, further clinical studies are needed to determine the benefits for patients (Horuk, 2009). Furthermore, long-term application of chemokine receptor inhibitors, e.g., CXCR4, is needed since considerable disadvantageous adverse reactions could occur in healthy tissue (Wang et al., 2016). Functional inhibition of CXCR4 can also cause stem cell mobilization, causing leukocytosis, thrombocytopenia, or spleen rupture when administered in a long-term manner (Wang et al., 2016; Kwon et al., 2019).

### Chimeric Antigen Receptor (CAR) T Cell Therapy

The function of CD8^+^ T cells is largely inhibited by the tumor and the surrounding tumor microenvironment. A promising approach is the use of CD8^+^ T cells to eliminate tumor cells by a so-called chimeric antigen receptor (CAR) T cell therapy. T cells are taken from a patient and transduced with a lentiviral vector to express a modified T cell receptor specifically recognizing TAAs. CAR-T cells are a complex of an Ig molecule and the T cell receptor. After *ex vivo* cell culture and stimulation, CAR-T cells are transferred back to the patient in order to eliminate tumor cells (Salinas et al., 2020). The advantage of CAR-T cells is that they are not dependent on MHC, as MHC expression is often downregulated in glioblastoma to hinder T cell activation (Fousek and Ahmed, 2015; Miyauchi and Tsirka, 2018). In a phase I study, recurrent glioblastoma and refractory glioblastoma were treated with CAR-T cells that have a high affinity to TAA interleukin-13 receptor α 2 (IL-13Rα2), which is overexpressed in recurrent glioblastoma. CAR-T cells were introduced into the tumor resection cavity, followed by infusions into the ventricular system of the brain. After administration the disease remission was sustained for 7.5 months with augmented levels of cytokines in one patient. It was suggested that recurrence occurs by decreased expression of IL-13R α2 (Brown et al., 2016; Dunn-Pirio and Vlahovic, 2017). One of the most severe complications in CAR-T cell treatment is cytokine release syndrome since inflammatory cytokines and chemokines are released, leading to nausea, headaches, tachycardia, hypotension, rashes, shortness of breath, and even multiorgan failure. Reversing the cytokine release syndrome with corticosteroids and anti-cytokine therapy is possible (Dunn-Pirio and Vlahovic, 2017). An alternative approach is the use of engineered CAR-NK cells to attack glioblastoma cells. However, CAR-NK cells demonstrated a minimal and unreliable response after application of NK cell lines. Primary NK cells from umbilical cord blood (UCB) could overcome this cavity and stimulation with IL-2 and IL-15 or UCB-derived NK cells expressing the TGF-β- dominant-negative receptor II, consuming TGF-β of the tumor microenvironment of glioblastoma is of interest (Burster et al., 2021).

### Vaccination to Induce Immune Responses Against Glioblastoma

A different study followed the idea of tumor vaccination to induce immune responses against specific antigens, peptides, DNA or mRNA-based (with or without vector) components. Vaccination could be used to target highly immunogenic TAAs or tumor-specific antigens to trigger a specific immune response. In this regard, glioblastoma care is challenging since tumor mutational load (burden) is rather low compared to other cancer types and glioblastoma patients have high variations of mutations. The tumor mutational burden correlates with the abundance of neoantigens, which can be potential biomarkers for immunotherapy (Hodges et al., 2017; Wang L. et al., 2020).

Rindopepimut—a peptide vaccine—targets EGFRvIII, which was administrated in combination with TMZ, displayed an improvement in early clinical trials, but did not reach the criteria for therapeutic efficacy in phase III (NCT01480479) (Malkki, 2016). The reappearance of the wildtype EGFR in recurrent glioblastoma precludes further treatment with Rindopepimut targeting EGFRvIII (Schuster et al., 2015). This mechanism is one immune evasion strategy by glioblastoma, which could be overcome by the use of vaccines directing homogenously expressed markers to tackle tumor cells, such as transformed IDH, targeting a broader range of TAAs (Phuphanich et al., 2013). For instance, a vaccine (IMA 950) directed 11 different human peptides (brevican; chondroitin sulfate proteoglycan 4; fatty acid binding protein 7; hepatitis B virus core antigen; insulin-like growth factor 2 messenger RNA-binding protein 3; neuronal cell adhesion molecule; protein tyrosine phosphatase, receptor-type, Z polypeptide 1; tenascin C; baculoviral inhibitor of apoptosis protein repeat-containing 5; Met proto-oncogene; neuroligin 4 X-linked). Of these, the first nine were previously identified on MHC I (human leukocyte antigen A^∗^02, HLA-A^∗^02), the other two are MHC II peptides derived from primary glioblastoma (Halford et al., 2014; Rampling et al., 2016). IMA 950 combined with poly-ICLC, a vaccine adjuvant enhancing innate and adaptive immune responses, revealed both CD4^+^ T cell and CD8^+^ T cell activation; with a median OS of 19 months (NCT01920191) (Migliorini et al., 2019). Peptide vaccines are specific for the respective tumor cells; however, peptide vaccines may only work for a small group of patients and might lead to immune evasion (Schneble et al., 2016). Targeting homogenously expressed targets or multi-peptide vaccines could interfere with immune evasion of tumor cells. Moreover, vaccination for glioblastoma management fail to sufficiently stimulate the immune system to achieve a clinical benefit, further approaches, e.g., combinational therapy, should be considered (Schneble et al., 2016; Lim et al., 2018).

In general, DC-based vaccines are generated by exposing DCs isolated from patients to the respective antigens and thereby educate DCs to maintain an adaptive immune response (Phuphanich et al., 2013). More precisely, DC-based vaccine ICT-07 targets six glioblastoma markers. Three of the six glioblastoma markers are human epidermal growth factor receptor 2 (HER2/neu), tyrosine-related protein 2, and absent in melanoma 2 which are also overexpressed in cancer stem-like cells (Phuphanich et al., 2013; Wen et al., 2019). Therefore, ICT-07 is thought to improve PFS and reduce the number of GSCs. Newly diagnosed glioblastoma patients had an increased OS and significantly extended PFS by 2.2 months in phase II. The vaccine was well tolerated with only a mild negative impact (due to insufficient financial resources, the phase III study is currently suspended, NCT02546102) (Phuphanich et al., 2013; Wen et al., 2019). Furthermore, a DC-based vaccine (DCVax^®^-L), where DCs are pulsed with tumor cell lysate and injected into the patient, is used for medication of newly diagnosed glioblastoma (NCT00045968). Phase I/II clinical trials determined the safety of the vaccine (Liau et al., 2018). 33% of patients with glioblastoma had a median OS of 48 months, 27% even achieved a median OS of 72 months in the long-term survival analysis, encouraging the use of DCVax^®^-L for glioblastoma therapy in the future (Dunn-Pirio and Vlahovic, 2017). Nevertheless, developing vaccines for individual neoantigens of patients is expensive and time-consuming because preparation of vaccines from the tumor samples take between 3–5 months (Peng et al., 2019). An additional limitation is the generation of sufficient DCs, as DCs comprise only < 1% of peripheral blood mononuclear cells. To overcome this obstacle, DCs were generated from monocytes *ex vivo*. However, it is questionable whether these monocyte-derived DCs compared to primary DCs from peripheral blood are efficient in an anti-tumor immune response (Huber et al., 2018). Furthermore, phagocytosis of tumor cells by APCs was enhanced by blocking the anti-phagocytosis molecule CD47 in combination with TMZ, inducing an effective anti-tumor immune response (von Roemeling et al., 2020). However, the use of whole tumor lysate to pulse DCs could cause autoimmune encephalitis since tumor lysate contains healthy brain tissue and induces an immune response toward the normal brain (Polyzoidis and Ashkan, 2014).

Moreover, a highly promising approach for cancer immunotherapy, denotes mRNA vaccine, which express tumor-specific antigens or TAA in APCs, has become into focus to treat glioblastoma. mRNA does not pose the risk of an infectious or an integrating agent, the potential of mRNA vaccines is the effectiveness, safety in administration, and low cost of manufacturing (Pardi et al., 2018; Weng et al., 2020). A phase I study utilizing DCs, loaded with TAA mRNA targeting cytomegalovirus pp65 protein that is expressed in > 90% of glioblastoma cases, demonstrated an OS of 35 months. As a consequence, co-delivery of mRNA vaccines together with immunotherapeutics can increase the host anti-tumor immune response (Batich et al., 2020; Miao et al., 2021). Notwithstanding the expected advantages, several factors limit the use of mRNA in therapy, including immunosuppressive effects of the tumor, half-life period of mRNA, and delivery complications *in vivo* (Vik-Mo et al., 2013; Weng et al., 2020). To overcome such issues, the chemical nucleotide modifications, capping analogs, and alternative delivery are currently being investigated and hold a great promise with current successful use of lipid nanoparticles to deliver mRNA vaccines (Weng et al., 2020; Rui and Green, 2021) or the use of viral vectors (Weng et al., 2020) and is therefore anticipated to increase the attention in glioblastoma immunotherapy.

### Heat Shock Protein (HSP) Vaccines

The exposure of environmental stress to cells leads to the production of HSPs. While HSPs act as chaperones, stabilizing protein conformation, and preventing protein aggregation, HSPs might also force misfolded proteins for degradation. Interestingly, a correlation between cancer and high levels of HSPs was determined, possibly by the excess of misfolded proteins found within the tumor (Ampie et al., 2015). On the one hand, gliomas overexpress HSP70 and HSP90 to prevent stress-induced apoptosis; on the other hand, HSPs have the ability to bind TAAs to elicit an immune response, making HSPs an interesting protein for immunotherapy (Ampie et al., 2015). The binding of HSPs to antigens can encounter the cell surface receptor CD91 of APCs in order to stimulate endocytosis and cross-present antigens to CD8^+^ T cells (Ampie et al., 2015). Although the application of an autologous HSP96-based vaccine (gp96-associated cellular peptides non-covalently bound to HSP96, HSPPC-96) in patients with recurrent glioblastoma are promising, this strategy is limited since the peptide pool is generated from the patient’s tumor and the amount of vaccine depends on the tumor size (Crane et al., 2013). Anti-inflammatory cytokines, such as IL-10 and TGF-β, segregated by the tumor microenvironment may also interfere with gp-96 vaccines and limits current clinical trials (Ampie et al., 2015; Li et al., 2020). Additionally, surgical resection is not always possible, the biopsy needs to be taken from glioblastoma patients to generate the vaccine, indicating the possibility of tumor progression (Bloch et al., 2014).

## Oncolytic Viruses in Immunotherapy

Myeloid cells are often activated by an oncolytic viral infection, which promotes an inflamed microenvironment by infiltration of T cells into the tumor. Therefore, oncolytic virotherapy is thought to be useful for overcoming the immunosuppressive environment in glioblastoma (Mogensen, 2009; Lim et al., 2018). Current oncolytic viral treatment applies replication-competent viruses rather than replication-incompetent viruses, explicitly adeno- and retroviruses, herpes simplex viruses, or measles- and polioviruses. Replication-competent viruses have the advantage of overcoming low transduction efficiency and vector loss. These viruses are genetically engineered to selectively infect and lyse cancer cells but the surrounding brain parenchyma is spared (Dunn-Pirio and Vlahovic, 2017; Foreman et al., 2017; Lim et al., 2018).

Oncolytic poliovirus originates from the oral poliovirus Sabin type I, which was genetically modified to replace its internal ribosome entry site with a human rhinovirus type 2 to eliminate neurovirulence. Oncolytic poliovirus infects cells expressing the poliovirus receptor (CD155), an oncofetal cell adhesion molecule, which is also expressed in glioblastoma, and subsequently in an immunogenic clearance of cancer cells (Desjardins et al., 2016; Mehta et al., 2017). Contrastingly, side effects of the viral treatment include cerebral edema as a result of a local inflammatory response and also known as pseudoprogression. Pseudoprogression is common in solid cancers after immunotherapy. In the case of brain malignancies, differentiation is difficult between pseudoprogression and tumor progression based on the neuroimages (Payer, 2011).

The replication-incompetent adenovirus is a tumoricidal gene vector. Aglatimagene besadenovec (AdV-tk) provokes the expression of the HSV-TK gene, which allows the conversion of prodrug ganciclovir or valacyclovir into a toxic nucleotide analog. This nucleotide analog kills replicating tumor cells by damaging the DNA and is also known as gene-mediated cytotoxic immunotherapy (Lim et al., 2018; Miyauchi and Tsirka, 2018). Two phase II clinical trials (BrTK02 and HGG-01) administered the virus intratumorally (BrTK02) or by intra-arterial cerebral infusion (HGG-01). Common detrimental effects were mostly fatigue, fever, and headaches (NCT00589875 for BrTK02 and NCT00870181 for HGG-01) (Chiocca et al., 2011; Ji et al., 2016; Wheeler et al., 2016). Nevertheless, non-replicating adenoviruses indicate limited distribution and low transduction efficiency of the vector (Ji et al., 2016). Furthermore, only a small amount of the injected adenovirus reaches the tumor due to elimination of the virus through the liver or by inactivation via binding to blood cells, complement, or to neutralizing antibodies, limiting further the successful application (Gao et al., 2014). The immune response represents an important factor for the efficiency of the drug. On the one hand, immune response limits the efficacy of oncolytic viruses, on the other hand, an anti-tumor immune response is crucial to fight against tumor cells.

## Combination Therapies Are More Effective in Treatment of Glioblastoma

Despite the expected potential of immunotherapy to treat glioblastoma, many monotherapy trials failed to reach sufficient efficiency. Besides the use of standard care of radiation/chemotherapy, combination therapies provide further enhancement in disease management (Dunn-Pirio and Vlahovic, 2017).

Adaptive resistance of tumor cells is an important factor contributing to the failure of monotherapy. Based on the nature of self-tolerance of the immune system, cancer cells can upregulate various immune checkpoint pathways to disturb the immune response (Medikonda et al., 2021). Notwithstanding that the use of monoclonal antibodies against these pathways might prevent inhibition of the immune response, phase II/III clinical studies targeting a single immune checkpoint pathway failed to be beneficial for glioblastoma patients (Majc et al., 2021; Medikonda et al., 2021). It is possible that immune checkpoint monotherapy forces PD-1-blockade resistance and upregulation of alternative immune checkpoint molecules, for instance, TIM-3 in a lung adenocarcinoma model (Koyama et al., 2016) may occur. While concurrent administration of anti-PD-1 and anti-TIM-3 antibodies improved preclinical glioblastoma, therapies with both antibodies and stereotactic radiosurgery resulted in 100% OS (Kim et al., 2017) and anti-PD-1 led to an enhanced vaccination-induced immune response (Antonios et al., 2016). Optimal timing of administration is important and concomitant treatment marks an optimal disease management (Lesterhuis et al., 2013). Other benefits of combining immune checkpoint inhibitors with standard care therapies or control of immune checkpoints, increase CD8^+^ T cell activity and decrease the infiltration of Treg cells (Huang et al., 2017). Bevacizumab in combination with common care (radio- and chemotherapy) remain below the expectation for immunotherapy since preclinical studies defined that high doses of TMZ lower the anti-PD-1 related immune response. Furthermore, the impact of chemotherapy to immunity is critical, for instance, it was not feasible to induce an antitumor response in mice treated with systemic chemotherapy when enabling a tumor re-challenge (Mathios et al., 2016; Lim et al., 2018).

Similarly, a combinatorial approach of different immunotherapies, including vaccines, immune checkpoint inhibitors, and effector lymphocytes, was verified for efficacy in glioblastoma treatment (Weenink et al., 2020). A recent study of CAR-T cell therapy combined with immune checkpoint blockade exhibited an enhanced tumor suppression effect compared to a single CAR-T construct in murine and canine models (Yin et al., 2018). Currently, clinical trials assessing the safety of CAR-T cells in combination with monoclonal antibodies (pembrolizumab, ipilimumab, and nivolumab; NCT03726515 and NCT04003649) are on the way. A different clinical trial, comprising neoantigen vaccination, indicated a systemic immune response and expression of multiple inhibitory checkpoints by infiltrating vaccine specific T cells, which was limited to individuals not receiving dexamethasone, suggesting a potential for combination of neoantigen vaccines with immune checkpoint inhibitors (Keskin et al., 2019). Nevertheless, severe side effects caused by the combination of nivolumab and ipilimumab (anti-CTLA-4) requires careful consideration of the possibility of disadvantageous reactions by combining different immunotherapies (Sampson et al., 2015).

## Further Complications in Glioblastoma

The 2019 severe acute respiratory syndrome coronavirus 2 (SARS-CoV-2) outbreak has brought its own significant obstacles to the care of patients suffering from glioblastoma. The pandemic disturbed the healthcare system leading to high hospital resource loads, risk of lack of treatment, and exposure to viral infection. Systemic immunosuppressive effects of standard anticancer medication, such as surgery and combined radio- and chemotherapy is a particular concern for glioblastoma patients. These patients are at a higher risk of infection and consequent complications (Amoo et al., 2020; Bernhardt et al., 2020; Noticewala et al., 2020).

**FIGURE 4:**
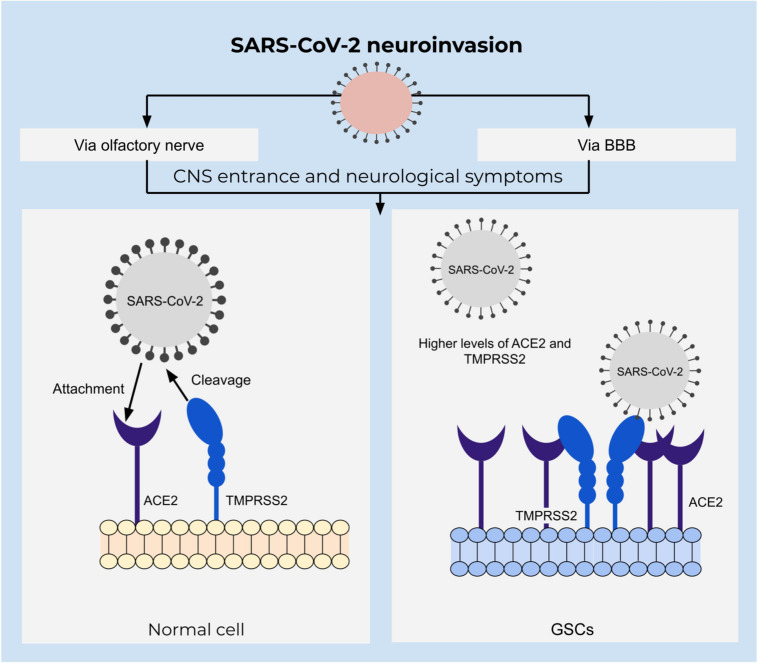
Integrative view of neuroinvasion and glioblastoma susceptibility to SARS-CoV-2. SARS-CoV-2 entrance depends on the attachment of the spike protein to the ACE2 receptor that is mediated by TMPRSS2 in a proteolytic manner. ACE2 is upregulated in GSCs and might increase susceptibility to SARS-CoV-2 infection.

SARS-CoV-2 belongs to a family of coronaviruses that are known as respiratory system pathogens, using their spike protein to bind cell surface receptors. In the case of SARS-CoV-2, this is mainly angiotensin-converting enzyme 2 (ACE2), which is a metallocarboxypeptidase present on surfaces of respiratory tract epithelial cells (Letko et al., 2020). Viral entrance is mediated by the serine protease transmembrane protease serine subtype 2 (TMPRSS2) that hydrolyze and prime the spike protein or alternatively via cathepsin B and cathepsin L in early endosomes (Wu et al., 2020). High abundance of ACE2 and TMPRSS2 in respiratory epithelial tissues explain the pathogenesis; however, expression of ACE2 is not limited to respiratory tract tissues, but is also found on brain cells (Figure 4). Moreover, an increasing number of obtained data on SARS-CoV-2 pathology indicate the presence of various neurological symptoms, such as fatigue, headaches, as well as smell- and taste impairments among more than a third of all the infected individuals (Mao et al., 2020). Cases of acute cerebrovascular disease with impaired consciousness suggests neural invasion of the virus (Conde Cardona et al., 2020; Mao et al., 2020). Several studies have identified the presence of the viral RNA in the central nervous system (Puelles et al., 2020; Meinhardt et al., 2021) as well as brain damage evidenced by neuroimaging studies (Coolen et al., 2020).

Accompanied by an increased vascular permeability caused by active chemokine secretion upon the viral infection, CNS becomes particularly susceptible to viral invasion (McGavern and Kang, 2011; Letko et al., 2020). An alternative route of CNS invasion was identified via the olfactory bulb and peripheral neurons, which was outlined to be the dominant pathway of entrance by the virus in transgenic mice (Wu et al., 2020; Song et al., 2021). Neurotropism and neural invasion of SARS-CoV-2 are defined by single cell transcriptome sequencing analysis of glioblastoma tissue (Wu et al., 2020). Moreover, an analysis of both U-87 and U-373 glioblastoma cell lines defined a susceptibility to infection and resistance to apoptosis. Additionally, these cells express ACE2, TMPRSS2, cathepsin B as well as cathepsin L which is important for entrance of SARS-CoV-2 to the target cell (Bielarz et al., 2021). An abundance of ACE2 receptors in glioblastoma tissue suggests glioblastoma patients to be at a particularly high risk of acquiring infection (Wu et al., 2020).

Preliminary findings from experimental studies on cancer patients prompt an involvement of tumor markers as additional sites for SARS-CoV-2 entry. One of the promising candidates is CD147 (also termed basigin) that was found to be overexpressed in glioblastoma tissue and positively correlated with the viral invasion. CD147 is involved in the assessment of T cells engineered to express CAR specific for CD147 (CD147-CART) treatment in an anticancer therapy under the phase I trial in patients with recurrent glioblastoma (NCT04045847) (Landras et al., 2019; Wang K. et al., 2020; Xia and Dubrovska, 2020). Despite the findings that CD147 is used for the entrance of SARS-CoV-2 to the target cell, these data are currently challenged (Shilts et al., 2021). While innovative insights have to be considered during the pandemic, tumor heterogeneity and immunosuppressive state are important in the development of more potent therapeutic applications for glioblastoma patients.

## Conclusion

In recent years, the debate of using immunotherapy in glioblastoma care has continuously been raised. The application of ADCs, peptides or mRNA vaccines, CAR-T cells and CAR-NK T cells, checkpoint and chemokine inhibitors, or oncolytic viruses are hopeful treatment constituents. Notwithstanding these encouraging strategies, only a few patients respond to immunotherapy, indicating the improvement of therapies or co-delivery of multiple immunotherapeutics to be successful in the management of glioblastoma. The complications can be attributed to immonoresistance mechanisms and the complex heterogenous nature of the tumor. Understanding the pathophysiological features of glioblastoma provides an emergence of different immunotherapy strategies, including the focus on a combination of medications and personalized approaches to sensitize glioblastoma to immunotherapies and improve the treatment outcome.

## Author Contributions

TB, RT, ZY, KM, M-AW, JB, and UK: writing, reviewing, and editing. All authors contributed to the article and approved the submitted version.

## Conflict of Interest

The authors declare that the research was conducted in the absence of any commercial or financial relationships that could be construed as a potential conflict of interest.

## Publisher’s Note

All claims expressed in this article are solely those of the authors and do not necessarily represent those of their affiliated organizations, or those of the publisher, the editors and the reviewers. Any product that may be evaluated in this article, or claim that may be made by its manufacturer, is not guaranteed or endorsed by the publisher.

## Abbreviations

ACE2: angiotensin-converting enzyme 2
ADCs: antibody-drug conjugates
AIF: apoptosis-inducing factor
AIM-2: absent in melanoma 2
APCs: antigen-presenting cells
ARG1: arginase
B7-H1: B7-homolog 1
Bak: Bcl-2 antagonistic killer
Bax: Bcl-2 associated X protein
Bcl-2: B cell lymphoma-2 protein
Bcl-Xl: Bcl-extra-long
BBB: blood-brain barrier
BH3: Bcl-2 homology domain 3
CAR: chimeric antigen receptor
CCR2: C-C chemokine receptor type 2
CD: cluster of differentiation
CNS: central nervous system
CRS: cytokine release syndrome
CSCs: cancer stem-like cells
CTLs: cytotoxic T lymphocytes
CTLA-4: cytotoxic T-lymphocyte -associated protein 4
CXCR4: C-X -C chemokine receptor type 4
DCs: dendritic cells
DNA: deoxyribonucleic acid
DNRII: dominant-negative TGF-beta receptor II
EGFR: epidermal growth factor receptor
EGFRvIII: epidermal growth factor receptor variant III
ECM: extracellular matrix
FcRn: neonatal Fc receptor
FDA: US Food and Drug Administration
G-CSF: granulocyte-colony stimulating factor
GAMs: glioma-associated microglia/macrophages
GDNF: glial-derived neurotrophic factor
GLUT: glucose transporter
GM-CSF: granulocyte-macrophage colony-stimulating factor
GSCs: glioblastoma stem cells
HER2/neu: human epidermal growth factor receptor 2
HGF/SF: hepatocyte growth factor/scatter factor
HIF-1: hypoxia-inducible factor 1
HSP: heat shock protein
HSV: herpes simplex virus
IAPs: inhibitors of apoptosis
IDO: indolamine 2,3-dioxygenase 1
IFN-γ: interferon-gamma
IKK: IκB kinase
IL: interleukin
IL-13Rα2: interleukin-13 receptor alpha 2
ILT/LIR: Ig-like transcript/leukocyte Ig-like receptors
KIR: killer-cell Ig-like receptors
mAb: monoclonal antibody
mTOR: mammalian target of rapamycin
mTORC2: mTOR complex 2
M-CSF: macrophage colony-stimulating factor
Mcl-1: myeloid cell leukemia factor 1
MCP-1: monocyte chemotactic protein 1
MDSCs: myeloid-derived suppressor cells
MDR-1: multidrug resistance protein 1
MGMT: methyl guanine methyl transferase
MHC I: major histocompatibility complex class I
MHC II: major histocompatibility complex class II
MMP: matrix metalloproteinase
MTIC: 3-methyl-(triazen-1-yl) imidazole-4-carboxamide
NFκB: nuclear factor kappa-light-chain-enhancer of activated B cells
NOS2: nitric oxide synthase 2
NSCLC: non-small cell lung cancer
OS: overall survival
P-gp: permeability glycoprotein
PD-1: programmed cell death protein 1
PD-L1: programmed death-ligand 1
PDK1: pyruvate dehydrogenase lipoamide kinase isozyme 1
PFS: progression-free survival
PI3K: phosphoinositide 3-kinase
PIP2: phosphatidylinositol 4,5-bisphosphate
PIP3: phosphatidylinositol (3,4,5)-trisphosphate
PTEN: phosphatase and tensin homolog
RNA: ribonucleic acid
ROS: reactive oxygen species
RTK: receptor tyrosine kinase
SARS-CoV-2: severe acute respiratory syndrome coronavirus 2
STAT3: signal transducer and activator of transcription 3
TAA: tumor-associated antigens
TAMs: tumor-associated macrophages
TGF-β: transforming growth factor-β
Th: T helper
TILs: tumor-infiltrating lymphocytes
TIM-3: T cell immunoglobulin and mucin domain-containing protein 3
TME: tumor microenvironment
TMPRSS2: transmembrane protease serine subtype 2
TMZ: temozolomide
TNF: tumor necrosis factor
TNFR1: tumor necrosis factor receptor 1
Treg: T regulatory cell
TRP-2: tyrosine-related protein 2
UCB: umbilical cord blood
VEGF: vascular endothelial growth factor.

## References

[B1] Adhikaree J. Moreno-Vicente J. Kaur A. P. Jackson A. M. Patel P. M. (2020). Resistance Mechanisms and Barriers to Successful Immunotherapy for Treating Glioblastoma. *Cells* 9:2. 10.3390/cells9020263 31973059 PMC7072315

[B2] Ahir B. K. Engelhard H. H. Lakka S. S. (2020). Tumor Development and Angiogenesis in Adult Brain Tumor: Glioblastoma. *Mol. Neurobiol.* 57 2461–2478. 10.1007/s12035-020-01892-8 32152825 PMC7170819

[B3] Amin M. M. Shawky A. Zaher A. Abdelbara M. Wasel Y. Gomaa M. (2012). Immune cell infiltrate in different grades of astrocytomas: possible role in the pathogenesis. *Egyptian J. Pathol.* 32:5.

[B4] Amoo M. Horan J. Gilmartin B. Nolan D. Corr P. MacNally S. (2020). The provision of neuro-oncology and glioma neurosurgery during the SARS-CoV-2 pandemic: a single national tertiary centre experience. *Ir. J. Med. Sci*. 2020:7. 10.1007/s11845-020-02429-7 33155104 PMC7643863

[B5] Ampie L. Choy W. Lamano J. B. Fakurnejad S. Bloch O. Parsa A. T. (2015). Heat shock protein vaccines against glioblastoma: from bench to bedside. *J. Neurooncol.* 123 441–448. 10.1007/s11060-015-1837-7 26093618 PMC4520407

[B6] An J. Chen Y. Huang Z. (2004). Critical upstream signals of cytochrome C release induced by a novel Bcl-2 inhibitor. *J. Biol. Chem.* 279 19133–19140. 10.1074/jbc.M400295200 14966123

[B7] Antonios J. P. Soto H. Everson R. G. Orpilla J. Moughon D. Shin N. (2016). PD-1 blockade enhances the vaccination-induced immune response in glioma. *JCI Insight* 1:10. 10.1172/jci.insight.87059 27453950 PMC4951098

[B8] Arora A. Somasundaram K. (2019). Glioblastoma vs temozolomide: can the red queen race be won? *Cancer Biol. Ther.* 20 1083–1090. 10.1080/15384047.2019.1599662 31068075 PMC6606031

[B9] Arslan F. Bosserhoff A. K. Nickl-Jockschat T. Doerfelt A. Bogdahn U. Hau P. (2007). The role of versican isoforms V0/V1 in glioma migration mediated by transforming growth factor-beta2. *Br. J. Cancer* 96 1560–1568. 10.1038/sj.bjc.6603766 17453002 PMC2359935

[B10] Badr C. E. Wurdinger T. Nilsson J. Niers J. M. Whalen M. Degterev A. (2011). Lanatoside C sensitizes glioblastoma cells to tumor necrosis factor-related apoptosis-inducing ligand and induces an alternative cell death pathway. *Neuro Oncol.* 13 1213–1224. 10.1093/neuonc/nor067 21757445 PMC3199161

[B11] Batich K. A. Mitchell D. A. Healy P. Herndon J. E. II Sampson J. H. (2020). Once, Twice, Three Times a Finding: Reproducibility of Dendritic Cell Vaccine Trials Targeting Cytomegalovirus in Glioblastoma. *Clin. Cancer Res.* 26 5297–5303. 10.1158/1078-0432.CCR-20-1082 32719000 PMC9832384

[B12] Bernhardt D. Wick W. Weiss S. E. Sahgal A. Lo S. S. Suh J. H. (2020). Neuro-oncology Management During the COVID-19 Pandemic With a Focus on WHO Grade III and IV Gliomas. *Neuro Oncol*. 2020:113. 10.1093/neuonc/noaa113 32369601 PMC7239150

[B13] Bhaduri A. Di Lullo E. Jung D. Muller S. Crouch E. E. Espinosa C. S. (2020). Outer Radial Glia-like Cancer Stem Cells Contribute to Heterogeneity of Glioblastoma. *Cell Stem Cell* 4:e46. 10.1016/j.stem.2019.11.015 31901251 PMC7029801

[B14] Bielarz V. Willemart K. Avalosse N. De Swert K. Lotfi R. Lejeune N. (2021). Susceptibility of neuroblastoma and glioblastoma cell lines to SARS-CoV-2 infection. *Brain Res.* 1758:147344. 10.1016/j.brainres.2021.147344 33556379 PMC7863753

[B15] Blahovcova E. Richterova R. Kolarovszki B. Dobrota D. Racay P. Hatok J. (2015). Apoptosis-related gene expression in tumor tissue samples obtained from patients diagnosed with glioblastoma multiforme. *Int. J. Mol. Med.* 36 1677–1684. 10.3892/ijmm.2015.2369 26459752

[B16] Bloch O. Crane C. A. Fuks Y. Kaur R. Aghi M. K. Berger M. S. (2014). Heat-shock protein peptide complex-96 vaccination for recurrent glioblastoma: a phase II, single-arm trial. *Neuro Oncol.* 16 274–279. 10.1093/neuonc/not203 24335700 PMC3895386

[B17] Brandes A. A. Bartolotti M. Tosoni A. Franceschi E. (2016). Nitrosoureas in the Management of Malignant Gliomas. *Curr. Neurol. Neurosci. Rep.* 16:13. 10.1007/s11910-015-0611-8 26750128

[B18] Brown C. E. Alizadeh D. Starr R. Weng L. Wagner J. R. Naranjo A. (2016). Regression of Glioblastoma after Chimeric Antigen Receptor T-Cell Therapy. *N. Engl. J. Med.* 375 2561–2569. 10.1056/NEJMoa1610497 28029927 PMC5390684

[B19] Burster T. Gartner F. Bulach C. Zhanapiya A. Gihring A. Knippschild U. (2021). Regulation of MHC I Molecules in Glioblastoma Cells and the Sensitizing of NK Cells. *Pharmaceuticals* 14:3. 10.3390/ph14030236 33800301 PMC7998501

[B20] Candeias S. M. Gaipl U. S. (2016). The Immune System in Cancer Prevention, Development and Therapy. *Anticancer. Agents Med. Chem.* 16 101–107. 10.2174/1871520615666150824153523 26299661

[B21] Carter N. J. Keating G. M. (2007). Maraviroc. *Drugs* 67 2277–2288. 10.2165/00003495-200767150-00010 17927288

[B22] Castriconi R. Cantoni C. Della Chiesa M. Vitale M. Marcenaro E. Conte R. (2003). Transforming growth factor beta 1 inhibits expression of NKp30 and NKG2D receptors: consequences for the NK-mediated killing of dendritic cells. *Proc Natl Acad Sci U S A* 100 4120–4125. 10.1073/pnas.0730640100 12646700 PMC153058

[B23] Chiocca E. A. Aguilar L. K. Bell S. D. Kaur B. Hardcastle J. Cavaliere R. (2011). Phase IB study of gene-mediated cytotoxic immunotherapy adjuvant to up-front surgery and intensive timing radiation for malignant glioma. *J. Clin. Oncol.* 29 3611–3619. 10.1200/JCO.2011.35.5222 21844505 PMC3179270

[B24] Conde Cardona G. Quintana Pajaro L. D. Quintero Marzola I. D. Ramos Villegas Y. Moscote Salazar L. R. (2020). Neurotropism of SARS-CoV 2: Mechanisms and manifestations. *J. Neurol. Sci.* 412:116824. 10.1016/j.jns.2020.116824 32299010 PMC7141641

[B25] Coolen T. Lolli V. Sadeghi N. Rovai A. Trotta N. Taccone F. S. (2020). Early postmortem brain MRI findings in COVID-19 non-survivors. *Neurology* 95 e2016–e2027. 10.1212/WNL.0000000000010116 32546654

[B26] Crane C. A. Han S. J. Ahn B. Oehlke J. Kivett V. Fedoroff A. (2013). Individual patient-specific immunity against high-grade glioma after vaccination with autologous tumor derived peptides bound to the 96 KD chaperone protein. *Clin. Cancer Res.* 19 205–214. 10.1158/1078-0432.CCR-11-3358 22872572

[B27] Darvin P. Toor S. M. Sasidharan Nair V. Elkord E. (2018). Immune checkpoint inhibitors: recent progress and potential biomarkers. *Exp. Mol. Med.* 50 1–11. 10.1038/s12276-018-0191-1 30546008 PMC6292890

[B28] De I. Steffen M. D. Clark P. A. Patros C. J. Sokn E. Bishop S. M. (2016). CSF1 Overexpression Promotes High-Grade Glioma Formation without Impacting the Polarization Status of Glioma-Associated Microglia and Macrophages. *Cancer Res.* 76 2552–2560. 10.1158/0008-5472.CAN-15-2386 27013192 PMC4873447

[B29] Dean M. Fojo T. Bates S. (2005). Tumour stem cells and drug resistance. *Nat. Rev. Cancer* 5 275–284. 10.1038/nrc1590 15803154

[B30] Desjardins A. Sampson J. H. Peters K. B. Vlahovic G. Randazzo D. Threatt S. (2016). Patient survival on the dose escalation phase of the Oncolytic Polio/Rhinovirus Recombinant (PVSRIPO) against WHO grade IV malignant glioma (MG) clinical trial compared to historical controls. *J. Clin. Oncol.* 34 2061–2061. 10.1200/JCO.2016.34.15_suppl.206127069080

[B31] Di Bari M. Tombolillo V. Conte C. Castigli E. Sciaccaluga M. Iorio E. (2015). Cytotoxic and genotoxic effects mediated by M2 muscarinic receptor activation in human glioblastoma cells. *Neurochem. Int.* 90 261–270. 10.1016/j.neuint.2015.09.008 26455407

[B32] Dunn-Pirio A. M. Vlahovic G. (2017). Immunotherapy approaches in the treatment of malignant brain tumors. *Cancer* 123 734–750. 10.1002/cncr.30371 27875627

[B33] Engelhardt B. Ransohoff R. M. (2012). Capture, crawl, cross: the T cell code to breach the blood-brain barriers. *Trends Immunol.* 33 579–589. 10.1016/j.it.2012.07.004 22926201

[B34] Foreman P. M. Friedman G. K. Cassady K. A. Markert J. M. (2017). Oncolytic Virotherapy for the Treatment of Malignant Glioma. *Neurotherapeutics* 14 333–344. 10.1007/s13311-017-0516-0 28265902 PMC5398989

[B35] Fousek K. Ahmed N. (2015). The Evolution of T-cell Therapies for Solid Malignancies. *Clin Cancer Res.* 21 3384–3392. 10.1158/1078-0432.CCR-14-2675 26240290 PMC4526112

[B36] Frankel S. R. (2003). Oblimersen sodium (G3139 Bcl-2 antisense oligonucleotide) therapy in Waldenstrom’s macroglobulinemia: a targeted approach to enhance apoptosis. *Semin. Oncol.* 30 300–304. 10.1053/sonc.2003.50041 12720157

[B37] Fricker S. P. (2008). A novel CXCR4 antagonist for hematopoietic stem cell mobilization. *Expert Opin. Investig. Drugs* 17 1749–1760. 10.1517/13543784.17.11.1749 18922110

[B38] Gabrusiewicz K. Rodriguez B. Wei J. Hashimoto Y. Healy L. M. Maiti S. N. (2016). Glioblastoma-infiltrated innate immune cells resemble M0 macrophage phenotype. *JCI Insight* 1:2. 10.1172/jci.insight.85841 26973881 PMC4784261

[B39] Gan H. K. van den Bent M. Lassman A. B. Reardon D. A. Scott A. M. (2017). Antibody-drug conjugates in glioblastoma therapy: the right drugs to the right cells. *Nat Rev Clin Oncol* 14 695–707. 10.1038/nrclinonc.2017.95 28675164

[B40] Gao Q. Chen C. Ji T. Wu P. Han Z. Fang H. (2014). A systematic comparison of the anti-tumoural activity and toxicity of the three Adv-TKs. *PLoS One* 9:e94050. 10.1371/journal.pone.0094050 24722669 PMC3983249

[B41] Gieryng A. Pszczolkowska D. Walentynowicz K. A. Rajan W. D. Kaminska B. (2017). Immune microenvironment of gliomas. *Lab. Invest.* 97 498–518. 10.1038/labinvest.2017.19 28287634

[B42] Halford S. Rampling R. James A. Peoples S. Mulholland P. Al-Salihi O. (2014). 1057PD - Final Results from a Cancer Research Uk First in Man Phase I Trial of Ima950 (A Novel Multi Peptide Vaccine) Plus Gm-Csf in Patients with Newly Diagnosed Glioblastoma. *Ann. Oncol.* 25:iv364. 10.1093/annonc/mdu342.10

[B43] Hanahan D. Weinberg R. A. (2011). Hallmarks of cancer: the next generation. *Cell* 144 646–674. 10.1016/j.cell.2011.02.013 21376230

[B44] Hegi M. E. Diserens A. C. Gorlia T. Hamou M. F. de Tribolet N. Weller M. (2005). MGMT gene silencing and benefit from temozolomide in glioblastoma. *N. Engl. J. Med.* 352 997–1003. 10.1056/NEJMoa043331 15758010

[B45] Herbener V. J. Burster T. Goreth A. Pruss M. von Bandemer H. Baisch T. (2020). Considering the Experimental use of Temozolomide in Glioblastoma Research. *Biomedicines* 8:151. 10.3390/biomedicines8060151 32512726 PMC7344626

[B46] Hira V. V. Verbovsek U. Breznik B. Srdic M. Novinec M. Kakar H. (2017). Cathepsin K cleavage of SDF-1alpha inhibits its chemotactic activity towards glioblastoma stem-like cells. *Biochim. Biophys. Acta Mol. Cell Res.* 1864 594–603. 10.1016/j.bbamcr.2016.12.021 28040478

[B47] Hlavac M. Dwucet A. Kast R. E. Engelke J. Westhoff M. A. Siegelin M. D. (2019). Combined inhibition of RAC1 and Bcl-2/Bcl-xL synergistically induces glioblastoma cell death through down-regulation of the Usp9X/Mcl-1 axis. *Cell Oncol.* 42 287–301. 10.1007/s13402-019-00425-3 30859392 PMC12994362

[B48] Hodges T. R. Ott M. Xiu J. Gatalica Z. Swensen J. Zhou S. (2017). Mutational burden, immune checkpoint expression, and mismatch repair in glioma: implications for immune checkpoint immunotherapy. *Neuro Oncol.* 19 1047–1057. 10.1093/neuonc/nox026 28371827 PMC5570198

[B49] Horuk R. (2009). Chemokine receptor antagonists: overcoming developmental hurdles. *Nat. Rev. Drug. Discov.* 8 23–33. 10.1038/nrd2734 19079127

[B50] Huang J. Liu F. Liu Z. Tang H. Wu H. Gong Q. (2017). Immune Checkpoint in Glioblastoma: promising and challenging. *Front. Pharmacol.* 8:242. 10.3389/fphar.2017.00242 28536525 PMC5422441

[B51] Huber A. Dammeijer F. Aerts J. Vroman H. (2018). Current State of Dendritic Cell-Based Immunotherapy: Opportunities for in vitro Antigen Loading of Different DC Subsets? *Front. Immunol.* 9:2804. 10.3389/fimmu.2018.02804 30559743 PMC6287551

[B52] Hussain S. F. Kong L. Y. Jordan J. Conrad C. Madden T. Fokt I. (2007). A novel small molecule inhibitor of signal transducers and activators of transcription 3 reverses immune tolerance in malignant glioma patients. *Cancer Res.* 67 9630–9636. 10.1158/0008-5472.CAN-07-1243 17942891

[B53] Jackson C. M. Choi J. Lim M. (2019). Mechanisms of immunotherapy resistance: lessons from glioblastoma. *Nat. Immunol.* 20 1100–1109. 10.1038/s41590-019-0433-y 31358997

[B54] Ji N. Weng D. Liu C. Gu Z. Chen S. Guo Y. (2016). Adenovirus-mediated delivery of herpes simplex virus thymidine kinase administration improves outcome of recurrent high-grade glioma. *Oncotarget* 7 4369–4378. 10.18632/oncotarget.6737 26716896 PMC4826211

[B55] Julien T. Frankel B. Longo S. Kyle M. Gibson S. Shillitoe E. (2000). Antisense-mediated inhibition of the bcl-2 gene induces apoptosis in human malignant glioma. *Surg. Neurol.* 53 360–368. 10.1016/s0090-3019(00)00178-6 10825522

[B56] Juszczak A. Gupta A. Karavitaki N. Middleton M. R. Grossman A. B. (2012). Ipilimumab: a novel immunomodulating therapy causing autoimmune hypophysitis: a case report and review. *Eur. J. Endocrinol.* 167 1–5. 10.1530/EJE-12-0167 22495490

[B57] Kaina B. (2019). Temozolomide in glioblastoma therapy: role of apoptosis, senescence and autophagy. comment on Strobel et al., temozolomide and other alkylating agents in glioblastoma therapy. Biomedicines 2019, 7, 69. *Biomedicines* 7:90. 10.3390/biomedicines7040090 31717973 PMC6966492

[B58] Kamson D. O. Grossman S. A. (2021). The Role of Temozolomide in Patients With Newly Diagnosed Wild-Type IDH, Unmethylated MGMTp Glioblastoma During the COVID-19 Pandemic. *JAMA Oncol.* 7 675–676. 10.1001/jamaoncol.2020.6732 33475680

[B59] Karachi A. Dastmalchi F. Mitchell D. A. Rahman M. (2018). Temozolomide for immunomodulation in the treatment of glioblastoma. *Neuro Oncol.* 20 1566–1572. 10.1093/neuonc/noy072 29733389 PMC6231207

[B60] Karpel-Massler G. Ishida C. T. Bianchetti E. Zhang Y. Shu C. Tsujiuchi T. (2017). Induction of synthetic lethality in IDH1-mutated gliomas through inhibition of Bcl-xL. *Nat. Commun.* 8:1067. 10.1038/s41467-017-00984-9 29057925 PMC5651864

[B61] Keskin D. B. Anandappa A. J. Sun J. Tirosh I. Mathewson N. D. Li S. (2019). Neoantigen vaccine generates intratumoral T cell responses in phase Ib glioblastoma trial. *Nature* 565 234–239. 10.1038/s41586-018-0792-9 30568305 PMC6546179

[B62] Kim J. E. Patel M. A. Mangraviti A. Kim E. S. Theodros D. Velarde E. (2017). Combination Therapy with Anti-PD-1, Anti-TIM-3, and Focal Radiation Results in Regression of Murine Gliomas. *Clin. Cancer Res.* 23 124–136. 10.1158/1078-0432.CCR-15-1535 27358487 PMC5735836

[B63] Knight M. J. Riffkin C. D. Muscat A. M. Ashley D. M. Hawkins C. J. (2001). Analysis of FasL and TRAIL induced apoptosis pathways in glioma cells. *Oncogene* 20 5789–5798. 10.1038/sj.onc.1204810 11593384

[B64] Kouri F. M. Jensen S. A. Stegh A. H. (2012). The role of Bcl-2 family proteins in therapy responses of malignant astrocytic gliomas: Bcl2L12 and beyond. *Sci. World J.* 2012:838916. 10.1100/2012/838916 22431925 PMC3289992

[B65] Koyama S. Akbay E. A. Li Y. Y. Herter-Sprie G. S. Buczkowski K. A. Richards W. G. (2016). Adaptive resistance to therapeutic PD-1 blockade is associated with upregulation of alternative immune checkpoints. *Nat. Commun.* 7:10501. 10.1038/ncomms10501 26883990 PMC4757784

[B66] Ku M. C. Wolf S. A. Respondek D. Matyash V. Pohlmann A. Waiczies S. (2013). GDNF mediates glioblastoma-induced microglia attraction but not astrogliosis. *Acta Neuropathol.* 125 609–620. 10.1007/s00401-013-1079-8 23344256

[B67] Kunkel P. Muller S. Schirmacher P. Stavrou D. Fillbrandt R. Westphal M. (2001). Expression and localization of scatter factor/hepatocyte growth factor in human astrocytomas. *Neuro Oncol.* 3 82–88. 10.1093/neuonc/3.2.82 11296484 PMC1920608

[B68] Kuter D. J. (2015). Managing thrombocytopenia associated with cancer chemotherapy. *Oncology* 29 282–294. 25952492

[B69] Kwon S. G. Park I. Kwon Y. W. Lee T. W. Park G. T. Kim J. H. (2019). Role of stem cell mobilization in the treatment of ischemic diseases. *Arch Pharm Res* 42 224–231. 10.1007/s12272-019-01123-2 30680545

[B70] Landras A. Reger, de Moura C. Jouenne F. Lebbe C. Menashi S. (2019). CD147 Is a Promising Target of Tumor Progression and a Prognostic Biomarker. *Cancers* 11:11. 10.3390/cancers11111803 31744072 PMC6896083

[B71] Laudati E. Curro D. Navarra P. Lisi L. (2017). Blockade of CCR5 receptor prevents M2 microglia phenotype in a microglia-glioma paradigm. *Neurochem Int* 108 100–108. 10.1016/j.neuint.2017.03.002 28279751

[B72] Le Rhun E. Preusser M. Roth P. Reardon D. A. van den Bent M. Wen P. (2019). Molecular targeted therapy of glioblastoma. *Cancer Treat Rev.* 80:101896. 10.1016/j.ctrv.2019.101896 31541850

[B73] Lesterhuis W. J. Salmons J. Nowak A. K. Rozali E. N. Khong A. Dick I. M. (2013). Synergistic effect of CTLA-4 blockade and cancer chemotherapy in the induction of anti-tumor immunity. *PLoS One* 8:e61895. 10.1371/journal.pone.0061895 23626745 PMC3633941

[B74] Letko M. Marzi A. Munster V. (2020). Functional assessment of cell entry and receptor usage for SARS-CoV-2 and other lineage B betacoronaviruses. *Nat. Microbiol.* 5 562–569. 10.1038/s41564-020-0688-y 32094589 PMC7095430

[B75] Li C. Du Y. Zhang Y. Ji N. (2020). Immunotherapy with heat shock protein 96 to treat gliomas. *Chin. Neurosurg. J.* 6:31. 10.1186/s41016-020-00211-3 32922959 PMC7469332

[B76] Li J. Y. Boado R. J. Pardridge W. M. (2001). Blood-brain barrier genomics. *J. Cereb. Blood Flow Metab.* 21 61–68. 10.1097/00004647-200101000-00008 11149669

[B77] Liau L. M. Ashkan K. Tran D. D. Campian J. L. Trusheim J. E. Cobbs C. S. (2018). First results on survival from a large Phase 3 clinical trial of an autologous dendritic cell vaccine in newly diagnosed glioblastoma. *J. Transl. Med.* 16 142. 10.1186/s12967-018-1507-6 29843811 PMC5975654

[B78] Lim M. Xia Y. Bettegowda C. Weller M. (2018). Current state of immunotherapy for glioblastoma. *Nat. Rev. Clin. Oncol.* 15 422–442. 10.1038/s41571-018-0003-5 29643471

[B79] Lincoln F. A. Imig D. Boccellato C. Juric V. Noonan J. Kontermann R. E. (2018). Sensitization of glioblastoma cells to TRAIL-induced apoptosis by IAP- and Bcl-2 antagonism. *Cell Death Dis.* 9:1112. 10.1038/s41419-018-1160-2 30385739 PMC6212537

[B80] Majc B. Novak M. Kopitar-Jerala N. Jewett A. Breznik B. (2021). Immunotherapy of Glioblastoma: Current Strategies and Challenges in Tumor Model Development. *Cells* 10:2. 10.3390/cells10020265 33572835 PMC7912469

[B81] Malkki H. (2016). Trial Watch: Glioblastoma vaccine therapy disappointment in Phase III trial. *Nat. Rev. Neurol.* 12:190. 10.1038/nrneurol.2016.38 27020557

[B82] Mao L. Jin H. Wang M. Hu Y. Chen S. He Q. (2020). Neurologic manifestations of hospitalized patients with coronavirus disease 2019 in Wuhan, China. *JAMA Neurol.* 77 683–690. 10.1001/jamaneurol.2020.1127 32275288 PMC7149362

[B83] Marvel D. Gabrilovich D. I. (2015). Myeloid-derived suppressor cells in the tumor microenvironment: expect the unexpected. *J. Clin. Invest.* 125 3356–3364. 10.1172/JCI80005 26168215 PMC4588239

[B84] Mathios D. Kim J. E. Mangraviti A. Phallen J. Park C. K. Jackson C. M. (2016). Anti-PD-1 antitumor immunity is enhanced by local and abrogated by systemic chemotherapy in GBM. *Sci. Transl. Med.* 8:370ra180. 10.1126/scitranslmed.aag2942 28003545 PMC5724383

[B85] McGavern D. B. Kang S. S. (2011). Illuminating viral infections in the nervous system. *Nat. Rev. Immunol.* 11 318–329. 10.1038/nri2971 21508982 PMC5001841

[B86] Medikonda R. Dunn G. Rahman M. Fecci P. Lim M. (2021). A review of glioblastoma immunotherapy. *J. Neurooncol.* 151 41–53. 10.1007/s11060-020-03448-1 32253714

[B87] Mehta A. M. Sonabend A. M. Bruce J. N. (2017). Convection-Enhanced Delivery. *Neurotherapeutics* 14 358–371. 10.1007/s13311-017-0520-4 28299724 PMC5398992

[B88] Meinhardt J. Radke J. Dittmayer C. Franz J. Thomas C. Mothes R. (2021). Olfactory transmucosal SARS-CoV-2 invasion as a port of central nervous system entry in individuals with COVID-19. *Nat. Neurosci.* 24 168–175. 10.1038/s41593-020-00758-5 33257876

[B89] Mercurio L. Ajmone-Cat M. A. Cecchetti S. Ricci A. Bozzuto G. Molinari A. (2016). Targeting CXCR4 by a selective peptide antagonist modulates tumor microenvironment and microglia reactivity in a human glioblastoma model. *J. Exp. Clin. Cancer Res.* 35:55. 10.1186/s13046-016-0326-y 27015814 PMC4807593

[B90] Miao L. Zhang Y. Huang L. (2021). mRNA vaccine for cancer immunotherapy. *Mol. Cancer* 20:41. 10.1186/s12943-021-01335-5 33632261 PMC7905014

[B91] Michels J. Johnson P. W. Packham G. (2005). Mcl-1. *Int. J. Biochem. Cell Biol.* 37 267–271. 10.1016/j.biocel.2004.04.007 15474972

[B92] Migliorini D. Dutoit V. Allard M. Grandjean Hallez N. Marinari E. Widmer V. (2019). Phase I/II trial testing safety and immunogenicity of the multipeptide IMA950/poly-ICLC vaccine in newly diagnosed adult malignant astrocytoma patients. *Neuro Oncol.* 21 923–933. 10.1093/neuonc/noz040 30753611 PMC6620642

[B93] Miyauchi J. T. Tsirka S. E. (2018). Advances in immunotherapeutic research for glioma therapy. *J. Neurol.* 265 741–756. 10.1007/s00415-017-8695-5 29209782 PMC5914508

[B94] Mogensen T. H. (2009). Pathogen recognition and inflammatory signaling in innate immune defenses. *Clin. Microbiol. Rev.* 22 240–273. 10.1128/CMR.00046-08 19366914 PMC2668232

[B95] Nguyen T. T. T. Ishida C. T. Shang E. Shu C. Torrini C. Zhang Y. (2019). Activation of LXRbeta inhibits tumor respiration and is synthetically lethal with Bcl-xL inhibition. *EMBO Mol. Med.* 11:e10769. 10.15252/emmm.201910769 31468706 PMC6783693

[B96] Noticewala S. S. Ludmir E. B. Bishop A. J. Chung C. Ghia A. J. Grosshans D. (2020). Radiation for Glioblastoma in the Era of Coronavirus Disease 2019 (COVID-19): Patient Selection and Hypofractionation to Maximize Benefit and Minimize Risk. *Adv. Radiat. Oncol.* 5 743–745. 10.1016/j.adro.2020.04.040 32775785 PMC7251361

[B97] Ola M. S. Nawaz M. Ahsan H. (2011). Role of Bcl-2 family proteins and caspases in the regulation of apoptosis. *Mol Cell Biochem* 351 41–58. 10.1007/s11010-010-0709-x 21210296

[B98] Ou A. Yung W. K. A. Majd N. (2020). Molecular Mechanisms of Treatment Resistance in Glioblastoma. *Int. J. Mol. Sci.* 22:1. 10.3390/ijms22010351 33396284 PMC7794986

[B99] Pardi N. Hogan M. J. Porter F. W. Weissman D. (2018). mRNA vaccines - a new era in vaccinology. *Nat. Rev. Drug. Discov.* 17 261–279. 10.1038/nrd.2017.243 29326426 PMC5906799

[B100] Payer F. (2011). Pseudoprogression oder Pseudorespons: Herausforderung an die Bildgebung des Glioblastoma multiforme. *Wiener Medizinische Wochenschrift* 161 13–19. 10.1007/s10354-010-0860-8 21312094

[B101] Peng M. Mo Y. Wang Y. Wu P. Zhang Y. Xiong F. (2019). Neoantigen vaccine: an emerging tumor immunotherapy. *Mol. Cancer* 18:128. 10.1186/s12943-019-1055-6 31443694 PMC6708248

[B102] Pessina F. Navarria P. Bellu L. Clerici E. Politi L. S. Tropeano M. P. (2020). Treatment of patients with glioma during the COVID-19 pandemic: what we learned and what we take home for the future. *Neurosurg. Focus* 49:E10. 10.3171/2020.9.FOCUS20704 33260137

[B103] Peters C. Brown S. (2015). Antibody-drug conjugates as novel anti-cancer chemotherapeutics. *Biosci. Rep.* 35:4. 10.1042/BSR20150089 26182432 PMC4613712

[B104] Phillips A. C. Boghaert E. R. Vaidya K. S. Mitten M. J. Norvell S. Falls H. D. (2016). ABT-414, an Antibody-Drug Conjugate Targeting a Tumor-Selective EGFR Epitope. *Mol. Cancer Ther.* 15 661–669. 10.1158/1535-7163.MCT-15-0901 26846818

[B105] Phuphanich S. Wheeler C. J. Rudnick J. D. Mazer M. Wang H. Nuno M. A. (2013). Phase I trial of a multi-epitope-pulsed dendritic cell vaccine for patients with newly diagnosed glioblastoma. *Cancer Immunol. Immunother.* 62 125–135. 10.1007/s00262-012-1319-0 22847020 PMC3541928

[B106] Polson A. G. Calemine-Fenaux J. Chan P. Chang W. Christensen E. Clark S. (2009). Antibody-drug conjugates for the treatment of non-Hodgkin’s lymphoma: target and linker-drug selection. *Cancer Res* 69 2358–2364. 10.1158/0008-5472.CAN-08-2250 19258515

[B107] Polyzoidis S. Ashkan K. (2014). DCVax(R)-L–developed by Northwest Biotherapeutics. *Hum. Vaccin. Immunother.* 10 3139–3145. 10.4161/hv.29276 25483653 PMC4514134

[B108] Portella L. Vitale R. De Luca S. D’Alterio C. Ierano C. Napolitano M. (2013). Preclinical development of a novel class of CXCR4 antagonist impairing solid tumors growth and metastases. *PLoS One* 8:e74548. 10.1371/journal.pone.0074548 24058588 PMC3772838

[B109] Puelles V. G. Lutgehetmann M. Lindenmeyer M. T. Sperhake J. P. Wong M. N. Allweiss L. (2020). Multiorgan and renal tropism of SARS-CoV-2. *N. Engl. J. Med.* 383 590–592. 10.1056/NEJMc2011400 32402155 PMC7240771

[B110] Pyonteck S. M. Akkari L. Schuhmacher A. J. Bowman R. L. Sevenich L. Quail D. F. (2013). CSF-1R inhibition alters macrophage polarization and blocks glioma progression. *Nat Med* 19 1264–1272. 10.1038/nm.3337 24056773 PMC3840724

[B111] Rampling R. Peoples S. Mulholland P. J. James A. Al-Salihi O. Twelves C. J. (2016). A Cancer Research UK First Time in Human Phase I Trial of IMA950 (Novel Multipeptide Therapeutic Vaccine) in Patients with Newly Diagnosed Glioblastoma. *Clin. Cancer Res.* 22 4776–4785. 10.1158/1078-0432.CCR-16-0506 27225692 PMC5026298

[B112] Razavi S. M. Lee K. E. Jin B. E. Aujla P. S. Gholamin S. Li G. (2016). Immune Evasion Strategies of Glioblastoma. *Front. Surg.* 3:11. 10.3389/fsurg.2016.00011 26973839 PMC4773586

[B113] Roberts H. C. Roberts T. P. Brasch R. C. Dillon W. P. (2000). Quantitative measurement of microvascular permeability in human brain tumors achieved using dynamic contrast-enhanced MR imaging: correlation with histologic grade. *AJNR Am. J. Neuroradiol.* 21 891–899. 10815665 PMC7976746

[B114] Rui Y. Green J. J. (2021). Overcoming delivery barriers in immunotherapy for glioblastoma. *Drug. Deliv. Transl. Res*. 2021:2. 10.1007/s13346-021-01008-2 34053034 PMC8164566

[B115] Salinas R. D. Durgin J. S. O’Rourke D. M. (2020). Potential of Glioblastoma-Targeted Chimeric Antigen Receptor (CAR) T-Cell Therapy. *CNS Drugs* 34 127–145. 10.1007/s40263-019-00687-3 31916100

[B116] Sampson J. H. Vlahovic G. Sahebjam S. Omuro A. M. P. Baehring J. M. Hafler D. A. (2015). Preliminary safety and activity of nivolumab and its combination with ipilimumab in recurrent glioblastoma (GBM): CHECKMATE-143. *J. Clin. Oncol.* 33 3010–3010. 10.1200/jco.2015.33.15_suppl.3010

[B117] Schneble E. Clifton G. T. Hale D. F. Peoples G. E. (2016). Peptide-Based Cancer Vaccine Strategies and Clinical Results. *Methods Mol. Biol.* 1403 797–817. 10.1007/978-1-4939-3387-7_46 27076168

[B118] Schneider S. W. Ludwig T. Tatenhorst L. Braune S. Oberleithner H. Senner V. (2004). Glioblastoma cells release factors that disrupt blood-brain barrier features. *Acta Neuropathol.* 107 272–276. 10.1007/s00401-003-0810-2 14730455

[B119] Schuster J. Lai R. K. Recht L. D. Reardon D. A. Paleologos N. A. Groves M. D. (2015). A phase II, multicenter trial of rindopepimut (CDX-110) in newly diagnosed glioblastoma: the ACT III study. *Neuro Oncol.* 17 854–861. 10.1093/neuonc/nou348 25586468 PMC4483122

[B120] Scotti C. Iamele L. Vecchia L. (2015). Antibody–drug conjugates: targeted weapons against cancer. *Antibody Technol. J.* 1:52914. 10.2147/ANTI.S52914

[B121] See A. P. Han J. E. Phallen J. Binder Z. Gallia G. Pan F. (2012). The role of STAT3 activation in modulating the immune microenvironment of GBM. *J. Neurooncol.* 110 359–368. 10.1007/s11060-012-0981-6 23096132 PMC3700337

[B122] Shang E. Nguyen T. T. T. Shu C. Westhoff M. A. Karpel-Massler G. Siegelin M. D. (2020). Epigenetic Targeting of Mcl-1 Is Synthetically Lethal with Bcl-xL/Bcl-2 Inhibition in Model Systems of Glioblastoma. *Cancers* 12:8. 10.3390/cancers12082137 32752193 PMC7464325

[B123] Sharifzad F. Ghavami S. Verdi J. Mardpour S. Mollapour Sisakht M. Azizi Z. (2019). Glioblastoma cancer stem cell biology: potential theranostic targets. *Drug Resist. Updat.* 42 35–45. 10.1016/j.drup.2018.03.003 30877905

[B124] Shilts J. Crozier T. W. M. Greenwood E. J. D. Lehner P. J. Wright G. J. (2021). No evidence for basigin/CD147 as a direct SARS-CoV-2 spike binding receptor. *Sci. Rep.* 11:413. 10.1038/s41598-020-80464-1 33432067 PMC7801465

[B125] Singh R. Letai A. Sarosiek K. (2019). Regulation of apoptosis in health and disease: the balancing act of BCL-2 family proteins. *Nat. Rev. Mol. Cell Biol.* 20 175–193. 10.1038/s41580-018-0089-8 30655609 PMC7325303

[B126] Song E. Zhang C. Israelow B. Lu-Culligan A. Prado A. V. Skriabine S. (2021). Neuroinvasion of SARS-CoV-2 in human and mouse brain. *J Exp Med* 218 3. 10.1084/jem.20202135 33433624 PMC7808299

[B127] Stegh A. H. Kim H. Bachoo R. M. Forloney K. L. Zhang J. Schulze H. (2007). Bcl2L12 inhibits post-mitochondrial apoptosis signaling in glioblastoma. *Genes Dev.* 21 98–111. 10.1101/gad.1480007 17210792 PMC1759904

[B128] Steinbach J. P. Weller M. (2004). Apoptosis in Gliomas: molecular Mechanisms and Therapeutic Implications. *J. Neurooncol.* 70 247–256. 10.1007/s11060-004-2753-4 15674482

[B129] Stepanenko A. A. Chekhonin V. P. (2019). On the critical issues in temozolomide research in glioblastoma: clinically relevant concentrations and MGMT-independent resistance. *Biomedicines* 7:92. 10.3390/biomedicines7040092 31783653 PMC6966644

[B130] Strepkos D. Markouli M. Klonou A. Piperi C. Papavassiliou A. (2020). Insights in the immunobiology of glioblastoma. *J. Mole. Med.* 98:4. 10.1007/s00109-019-01835-4 31650201

[B131] Strik H. Deininger M. Streffer J. Grote E. Wickboldt J. Dichgans J. (1999). BCL-2 family protein expression in initial and recurrent glioblastomas: modulation by radiochemotherapy. *J. Neurol. Neurosurg. Psychiatr.* 67 763–768. 10.1136/jnnp.67.6.763 10567494 PMC1736652

[B132] Strobel H. Baisch T. Fitzel R. Schilberg K. Siegelin M. D. Karpel-Massler G. (2019). Temozolomide and Other Alkylating Agents in Glioblastoma Therapy. *Biomedicines* 7:3. 10.3390/biomedicines7030069 31505812 PMC6783999

[B133] Tagscherer K. E. Fassl A. Campos B. Farhadi M. Kraemer A. Bock B. C. (2008). Apoptosis-based treatment of glioblastomas with ABT-737, a novel small molecule inhibitor of Bcl-2 family proteins. *Oncogene* 27 6646–6656. 10.1038/onc.2008.259 18663354

[B134] Tron A. E. Belmonte M. A. Adam A. Aquila B. M. Boise L. H. Chiarparin E. (2018). Discovery of Mcl-1-specific inhibitor AZD5991 and preclinical activity in multiple myeloma and acute myeloid leukemia. *Nat. Commun.* 9:5341. 10.1038/s41467-018-07551-w 30559424 PMC6297231

[B135] Tsujimoto Y. (1998). Role of Bcl-2 family proteins in apoptosis: apoptosomes or mitochondria? *Genes Cells* 3 697–707. 10.1046/j.1365-2443.1998.00223.x 9990505

[B136] Tyagi D. Sharma B. S. Gupta S. K. Kaul D. Vasishta R. K. Khosla V. K. (2002). Expression of Bcl2 proto-oncogene in primary tumors of the central nervous system. *Neurol. India* 50 290–294. 12391455

[B137] van den Bent M. Gan H. K. Lassman A. B. Kumthekar P. Merrell R. Butowski N. (2017). Efficacy of depatuxizumab mafodotin (ABT-414) monotherapy in patients with EGFR-amplified, recurrent glioblastoma: results from a multi-center, international study. *Cancer Chemother. Pharmacol.* 80 1209–1217. 10.1007/s00280-017-3451-1 29075855 PMC5686264

[B138] Vellanki S. H. Grabrucker A. Liebau S. Proepper C. Eramo A. Braun V. (2009). Small-molecule XIAP inhibitors enhance gamma-irradiation-induced apoptosis in glioblastoma. *Neoplasia* 11 743–752. 10.1593/neo.09436 19649204 PMC2713593

[B139] Vengoji R. Macha M. A. Batra S. K. Shonka N. A. (2018). Natural products: a hope for glioblastoma patients. *Oncotarget* 9 22194–22219. 10.18632/oncotarget.25175 29774132 PMC5955138

[B140] Vieira de Castro J. Goncalves C. S. Hormigo A. Costa B. M. (2020). Exploiting the Complexities of Glioblastoma Stem Cells: Insights for Cancer Initiation and Therapeutic Targeting. *Int. J. Mol. Sci.* 21:15. 10.3390/ijms21155278 32722427 PMC7432229

[B141] Viel S. Marcais A. Guimaraes F. S. Loftus R. Rabilloud J. Grau M. (2016). TGF-beta inhibits the activation and functions of NK cells by repressing the mTOR pathway. *Sci. Signal* 9:ra19. 10.1126/scisignal.aad1884 26884601

[B142] Vik-Mo E. O. Nyakas M. Mikkelsen B. V. Moe M. C. Due-Tonnesen P. Suso E. M. (2013). Therapeutic vaccination against autologous cancer stem cells with mRNA-transfected dendritic cells in patients with glioblastoma. *Cancer Immunol. Immunother.* 62 1499–1509. 10.1007/s00262-013-1453-3 23817721 PMC3755221

[B143] von Roemeling C. A. Wang Y. Qie Y. Yuan H. Zhao H. Liu X. (2020). Therapeutic modulation of phagocytosis in glioblastoma can activate both innate and adaptive antitumour immunity. *Nat Commun* 11 1508. 10.1038/s41467-020-15129-8 32198351 PMC7083893

[B144] Wang H. B. Li T. Ma D. Z. Ji Y. X. Zhi H. (2017). Overexpression of FADD and Caspase-8 inhibits proliferation and promotes apoptosis of human glioblastoma cells. *Biomed. Pharmacother.* 93 1–7. 10.1016/j.biopha.2017.05.105 28618251

[B145] Wang K. Chen W. Zhou Y.-S. Lian J.-Q. Zhang Z. Du P. (2020). SARS-CoV-2 invades host cells via a novel route: CD147-spike protein. *bioRxiv* 2020:988345. 10.1101/2020.03.14.988345

[B146] Wang L. Ge J. Lan Y. Shi Y. Luo Y. Tan Y. (2020). Tumor mutational burden is associated with poor outcomes in diffuse glioma. *BMC Cancer* 20:213. 10.1186/s12885-020-6658-1 32164609 PMC7069200

[B147] Wang Y. Xie Y. Oupicky D. (2016). Potential of CXCR4/CXCL12 Chemokine Axis in Cancer Drug Delivery. *Curr. Pharmacol. Rep.* 2 1–10. 10.1007/s40495-015-0044-8 27088072 PMC4827436

[B148] Wängler B. Schirrmacher R. Wängler C. (2020). Aiming at the tumor-specific accumulation of MGMT-inhibitors: first description of a synthetic strategy towards inhibitor-peptide conjugates. *Tetrahed. Lett.* 61:151840. 10.1016/j.tetlet.2020.151840

[B149] Weathers S. P. de Groot J. (2015). VEGF Manipulation in Glioblastoma. *Oncology* 29 720–727. 26470893

[B150] Wee B. Pietras A. Ozawa T. Bazzoli E. Podlaha O. Antczak C. (2016). ABCG2 regulates self-renewal and stem cell marker expression but not tumorigenicity or radiation resistance of glioma cells. *Sci. Rep.* 6:25956. 10.1038/srep25956 27456282 PMC4960591

[B151] Weenink B. French P. J. Sillevis Smitt P. A. E. Debets R. Geurts M. (2020). Immunotherapy in Glioblastoma: current shortcomings and future perspectives. *Cancers* 12:3. 10.3390/cancers12030751 32235752 PMC7140029

[B152] Wen P. Y. Reardon D. A. Armstrong T. S. Phuphanich S. Aiken R. D. Landolfi J. C. (2019). A Randomized Double-Blind Placebo-Controlled Phase II Trial of Dendritic Cell Vaccine ICT-107 in Newly Diagnosed Patients with Glioblastoma. *Clin Cancer Res* 25 5799–5807. 10.1158/1078-0432.CCR-19-0261 31320597 PMC8132111

[B153] Weng Y. Li C. Yang T. Hu B. Zhang M. Guo S. (2020). The challenge and prospect of mRNA therapeutics landscape. *Biotechnol. Adv.* 40:107534. 10.1016/j.biotechadv.2020.107534 32088327

[B154] Westhoff M. A. Baisch T. Herbener V. J. Karpel-Massler G. Debatin K. M. Strobel H. (2020). Comment in response to “temozolomide in glioblastoma therapy: role of apoptosis, senescence and autophagy etc. by B. Kaina”. *Biomedicines* 8:93. 10.3390/biomedicines8040093 32326020 PMC7235879

[B155] Wheeler L. A. Manzanera A. G. Bell S. D. Cavaliere R. McGregor J. M. Grecula J. C. (2016). Phase II multicenter study of gene-mediated cytotoxic immunotherapy as adjuvant to surgical resection for newly diagnosed malignant glioma. *Neuro Oncol.* 18 1137–1145. 10.1093/neuonc/now002 26843484 PMC4933478

[B156] Wick W. Wild-Bode C. Frank B. Weller M. (2004). BCL-2-induced glioma cell invasiveness depends on furin-like proteases. *J. Neurochem.* 91 1275–1283. 10.1111/j.1471-4159.2004.02806.x 15584904

[B157] Wiendl H. Mitsdoerffer M. Hofmeister V. Wischhusen J. Bornemann A. Meyermann R. (2002). A functional role of HLA-G expression in human gliomas: an alternative strategy of immune escape. *J. Immunol.* 168 4772–4780. 10.4049/jimmunol.168.9.4772 11971028

[B158] Wilson W. H. O’Connor O. A. Czuczman M. S. LaCasce A. S. Gerecitano J. F. Leonard J. P. (2010). Navitoclax, a targeted high-affinity inhibitor of BCL-2, in lymphoid malignancies: a phase 1 dose-escalation study of safety, pharmacokinetics, pharmacodynamics, and antitumour activity. *The Lancet Oncol.* 11 1149–1159. 10.1016/s1470-2045(10)70261-8 21094089 PMC3025495

[B159] Wu A. Wei J. Kong L. Y. Wang Y. Priebe W. Qiao W. (2010). Glioma cancer stem cells induce immunosuppressive macrophages/microglia. *Neuro Oncol.* 12 1113–1125. 10.1093/neuonc/noq082 20667896 PMC3098021

[B160] Wu B. Wang W. Wang H. Zou Q. Hu B. Ye L. (2020). Single-Cell Sequencing of Glioblastoma Reveals Central Nervous System Susceptibility to SARS-CoV-2. *Front. Oncol.* 10:566599. 10.3389/fonc.2020.566599 33312949 PMC7703438

[B161] Xia P. Dubrovska A. (2020). Tumor markers as an entry for SARS-CoV-2 infection? *FEBS J.* 287 3677–3680. 10.1111/febs.15499 32738184 PMC7436716

[B162] Xiang W. Yang C. Y. Bai L. (2018). MCL-1 inhibition in cancer treatment. *Onco Targets Ther.* 11 7301–7314. 10.2147/OTT.S146228 30425521 PMC6205821

[B163] Xie Q. Mittal S. Berens M. E. (2014). Targeting adaptive glioblastoma: an overview of proliferation and invasion. *Neuro Oncol.* 16 1575–1584. 10.1093/neuonc/nou147 25082799 PMC4232088

[B164] Yin Y. Boesteanu A. C. Binder Z. A. Xu C. Reid R. A. Rodriguez J. L. (2018). Checkpoint Blockade Reverses Anergy in IL-13Ralpha2 Humanized scFv-Based CAR T Cells to Treat Murine and Canine Gliomas. *Mol. Ther. Oncolytics* 11 20–38. 10.1016/j.omto.2018.08.002 30306125 PMC6174845

[B165] Yu W. Zhang L. Wei Q. Shao A. (2020). O(6)-methylguanine-DNA methyltransferase (MGMT): challenges and new opportunities in glioma chemotherapy. *Front. Oncol.* 9:1547. 10.3389/fonc.2019.01547 32010632 PMC6979006

[B166] Zhang Q. Liu F. (2020). Advances and potential pitfalls of oncolytic viruses expressing immunomodulatory transgene therapy for malignant gliomas. *Cell Death Dis.* 11:485. 10.1038/s41419-020-2696-5 32587256 PMC7316762

